# National Trends in the Prevalence of Self-Perceived Overweight Among Adolescents Between 2005 and 2022: Nationwide Representative Study

**DOI:** 10.2196/57803

**Published:** 2024-10-09

**Authors:** Jinyoung Jeong, Seungjun Lee, Kyeongmin Lee, Seokjun Kim, Jaeyu Park, Yejun Son, Hyeri Lee, Hayeon Lee, Jiseung Kang, Masoud Rahmati, Damiano Pizzol, Lee Smith, Guillermo F López Sánchez, Elena Dragioti, Guillaume Fond, Laurent Boyer, Selin Woo, Sang Youl Rhee, Dong Keon Yon

**Affiliations:** 1 Department of Medicine Kyung Hee University College of Medicine Seoul Republic of Korea; 2 Center for Digital Health Medical Science Research Institute Kyung Hee University College of Medicine Seoul Republic of Korea; 3 Department of Regulatory Science Kyung Hee University Seoul Republic of Korea; 4 Department of Precision Medicine Kyung Hee University College of Medicine Seoul Republic of Korea; 5 Division of Sleep Medicine Harvard Medical School Boston, MA United States; 6 Department of Anesthesia Critical Care and Pain Medicine Massachusetts General Hospital Boston, MA United States; 7 Department of Physical Education and Sport Sciences Faculty of Literature and Human Sciences Lorestan University Khoramabad Iran; 8 Department of Physical Education and Sport Sciences Faculty of Literature and Humanities Vali-E-Asr University of Rafsanjan Rafsanjan Iran; 9 CEReSS-Health Service Research and Quality of Life Center Assistance Publique-Hôpitaux de Marseille Aix-Marseille University Marseille France; 10 Health Unit Eni San Donato Milanese Italy; 11 Health Unit Eni Maputo Mozambique; 12 Centre for Health, Performance, and Wellbeing Anglia Ruskin University Cambridge United Kingdom; 13 Division of Preventive Medicine and Public Health Department of Public Health Sciences School of Medicine, University of Murcia Murica Spain; 14 Research Laboratory Psychology of Patients, Families and Health Professionals Department of Nursing School of Health Sciences, University of Ioannina Ioannina Greece; 15 Department of Endocrinology and Metabolism Kyung Hee University School of Medicine Seoul Republic of Korea; 16 Department of Pediatrics Kyung Hee University Medical Center Kyung Hee University College of Medicine Seoul Republic of Korea

**Keywords:** self-perceived overweight, trend, prevalence, South Korea, adolescent

## Abstract

**Background:**

Despite several studies on self-evaluation of health and body shape, existing research on the risk factors of self-perceived overweight is insufficient, especially during the COVID-19 pandemic.

**Objective:**

This study aims to identify the risk factors affecting self-perceived overweight and examine how the prevalence of self-perceived overweight has changed before and during the COVID-19 pandemic. Specifically, we analyzed the impact of altered lifestyles due to COVID-19 on this phenomenon.

**Methods:**

The data used in the study were obtained from middle and high school students who participated in the Korean Youth Risk Behavior Web-based Survey (N=1,189,586). This survey was a 2-stage stratified cluster sampling survey representative of South Korean adolescents. We grouped the survey results by year and estimated the slope in the prevalence of self-perceived overweight before and during the pandemic using weighted linear regression, as well as the prevalence tendencies of self-perceived overweight according to various risk factors. We used prevalence ratios to identify the risk factors for self-perceived overweight. In addition, we conducted comparisons of risk factors in different periods to identify their associations with the COVID-19 pandemic.

**Results:**

The prevalence of self-perceived overweight was much higher than BMI-based overweight among 1,189,586 middle and high school participants (grade 7-12) from 2005 to 2022 (female participants: n=577,102, 48.51%). From 2005 to 2019 (prepandemic), the prevalence of self-perceived overweight increased (β=2.80, 95% CI 2.70-2.90), but from 2020 to 2022 (pandemic) it decreased (β=–0.53, 95% CI –0.74 to –0.33). During the pandemic, individuals with higher levels of stress or lower household economic status exhibited a more substantial decrease in the rate of self-perceived overweight. The prevalence of self-perceived overweight tended to be higher among individuals with poor academic performance, lower economic status, poorer subjective health, and a higher stress level.

**Conclusions:**

Our nationwide study, conducted over 18 years, indicated that self-perceived overweight decreased during the COVID-19 period while identifying low academic performance and economic status as risk factors. These findings suggest the need for policies and facilities to address serious dieting and body dissatisfaction resulting from self-perceived overweight by developing counseling programs for adolescents with risk factors such as lower school performance and economic status.

## Introduction

### Background

Amid the ongoing global health crisis, the psychological implications of the COVID-19 pandemic on body image perception need to be investigated [1]. The World Health Organization defines *overweight* as having a BMI ≥25 kg/m^2^ and *obesity* as having a BMI ≥30 kg/m^2^. The proportion of adolescents meeting these criteria ranged from 5.6% for girls to 7.8% for boys globally from 1975 to 2016 [2,3]. Despite these clear medical guidelines, individuals’ perceptions of weight can diverge substantially from these standards [4].

Self-perceived overweight refers to individuals who consider themselves to be a bit fat or very fat, regardless of the World Health Organization’s BMI standards for overweight. This perception potentially results in rigorous dieting, body dissatisfaction, and eating disorders [5,6]. Rigorous dieting can lead to nutritional deficiencies and impaired growth, body dissatisfaction is linked to low self-esteem and depression, and eating disorders can have severe physical and psychological effects, including increased mortality risk. In addition, these issues are rapidly escalating and are becoming broader public health concerns [7]. The long-term effects of body dissatisfaction that begin during adolescence can extend into adulthood, increasing the risk of chronic health issues, such as obesity, cardiovascular diseases, and persistent mental health-related disorders [8]. Therefore, it is essential to develop preventive strategies through research to mitigate these negative outcomes.

Nevertheless, prior research exploring body dissatisfaction has been limited by small sample sizes that challenge the veracity of findings (n=498) and do not encompass the COVID-19 pandemic era, hence lacking data to assess the impact of this pandemic on self-perceived overweight [6]. However, our study spans a long period and analyzes large-scale data, including the risk factors for self-perceived overweight during the COVID-19 era. Particularly, the COVID-19 period is significant as it brought about various changes to daily life, adversely affecting depression, anxiety, and stress levels [9]. This context enhances the relevance and urgency of our study, addressing the gap in understanding how the pandemic influenced self-perceived overweight among adolescents.

### Objectives

There is a pressing need to analyze the prevalence of self-perceived overweight among adolescents, including in the COVID-19 pandemic era, and to identify risk factors. Our study aimed to evaluate the prevalence of adolescents who are self-perceived overweight, including trends observed during the COVID-19 pandemic, and identify the associated risk factors. This study has the potential to propose evidence-based interventions and policies to support the mental and physical health of adolescents. In addition, the results of this study could help create targeted public health initiatives aimed at promoting a positive body image and preventing adverse effects.

## Methods

### Patient Selection and Data Collection

In this study, we used the nationwide Korea Youth Risk Behavior Web-based Survey (KYRBS) over 18 years from 2005 to 2022 to investigate the prevalence of self-perceived overweight among adolescents [10]. The KYRBS is an annual survey conducted by the Korea Disease Control and Prevention Agency and the Ministry of Education to examine the health behavior statistics of the youth in Korea [10]. The KYRBS survey includes categories, such as smoking, drinking, and obesity, and students voluntarily participate in an anonymous web survey. Our study focused on students in grades 7th to 12th from 800 schools, aged between 13 and 18 years, with an average response rate of >95%. This sample was extracted through the following 3 stages: population stratification, sampling distribution, and stratified cluster sampling [11]. In the population stratification stage, the population was divided into 117 strata based on 39 regional groups and schools to minimize sampling errors. In the sampling distribution stage, the size of the regional groups and types of schools were considered in selecting schools for the survey to ensure the composition of the population matched that of the sample. In the stratified cluster sampling stage, 2 rounds of extraction (at the school and classroom levels) were conducted, and all students in the selected classrooms were chosen as participants of the survey.

### Ascertainment of Considering Self-Perceived Overweight

The purpose of the study was to examine the proportion and trend of people who consider themselves overweight among adolescents in South Korea, spanning from 2005 to 2022, including the era of the COVID-19 pandemic. To examine the number of students who reported self-perception of being overweight, the students were asked, “How would you describe your body type?” with 5 multiple-choice options: very thin, bit thin, average, bit fat, and very fat. The 2 options, bit fat and very fat, were combined to form the category “self-perceived overweight.” This was determined by referring to the criteria of the previous study; categorized individuals who perceived that their weight was higher than their right weight, considered themselves to be overweight [12].

### Covariates

In total, 11 covariates were used in the analysis: grade (7th-9th grade: middle school and 10th-12th grade: high school) [13], sex, region of residence (urban and rural), BMI group (underweight, normal, overweight, and obese) [13-16], school performance (high, middle-high, middle, middle-low, and low), stress level (high, middle-high, middle, middle-low, and low) [17], subjective health status (very healthy, healthy, normal, and unhealthy), smoking status within 1 month of the survey, alcohol consumption within 1 month of the survey [11], and economic status of households (high, middle-high, middle, middle-low, and low) [18]. These covariates were selected based on previous trend analysis studies that either addressed similar dependent variables to our study or were conducted on the same adolescent group to identify the association between self-perceived overweight and various risk factors, which is the objective of our study [13,17]. BMI was calculated as weight in kilograms divided by height in meters squared based on students’ self-reported weight and height. Following the 2017 Korean National Growth Charts, BMI was divided into 4 groups: underweight (<5th percentile), normal (5th-84th percentile), overweight (85th-94th percentile), and obese (≥95th percentile) [18]. Meanwhile, “BMI-based overweight” was determined by including both the overweight and obese categories within the BMI groups. It was compared with “self-perceived overweight” to identify the discrepancies between actual overweight prevalence and self-perceived overweight prevalence among adolescents. School performance, stress level, and economic status of households were estimated from self-reports of the participants to the following questions: “Over the past 12 months, how has your school performance been?” “How much stress do you typically experience?” and “What is the economic status of your household?” Multiple choices for these questions were provided as follows: low (<20th percentile), middle-low (20th-39th percentile), middle (40th-59th percentile), middle-high (60th-79th percentile), and high (≥80th percentile). The definitions of all covariates were derived from established, peer-reviewed literature [13].

### Statistical Analyses

This study presented unweighted crude analysis results as frequencies and proportions to represent the overall characteristics of the study population. In contrast, the weighted composite sample analysis with weights provided by the Korea Disease Control and Prevention Agency was expressed using weighted percentages and 95% CIs for the results. The KYRBS provides a unified weight derived from 3 primary rates: the sampling rate, the response rate, and the poststratification rate. First, the sampling rate is calculated to correct for selection bias by school and class. The response rate ensures that sampled students represent the population in each grade. Finally, the poststratification rate adjusts the weight to ensure that the sum matches the total student population. These rates collectively contribute to a weight that allows the KYRBS to accurately represent the population of middle and high school students in South Korea. The prevalence of self-perceived overweight was calculated by grouping KYRBS data classified by year from 2005 to 2022 into 3-year intervals. The prevalence of self-perceived overweight and all regression model analyses were calculated, considering various variables, such as grade, sex, region of residence, BMI, school performance, stress level, subjective health status, smoking status, alcohol consumption, and economic status of households. To calculate the 95% CI for the β coefficient, linear regression models were used [12,13,17]. Weighted prevalence ratios (wPRs) were used to identify risk factors among the independent variables. The risk for a specific case is expressed using prevalence ratios relative to a reference case. Meanwhile, linear regression is a regression method used to identify the relationship between a dependent variable and 1 or more independent variables. We used this model to examine the trend of self-perceived overweight over the years. The trend in self-perceived overweight according to the year groups, set as the independent variable, was represented by the β-coefficients of the model. We computed both the Cox and Snell *R*^2^ and the Nagelkerke (max-rescaled) *R*^2^- values in our regression model, adjusted for all covariates, which are specifically designed for logistic regression to assess a variance explanation of the model [19,20]. Our model showed an explanatory power from Cox and Snell *R*^2^ of 0.1562 and a Nagelkerke *R*^2^ of 0.2142 [21]. Moreover, the model’s ability to classify outcomes is further calculated by the area under the receiver operating characteristic curve. Our model achieved an area under the receiver operating characteristic curve of 0.7447, confirming its notable performance [22]. In this study, 95% CI values for β-coefficients were computed for analysis of trends in both prepandemic and during the pandemic, and β difference (β_diff_) was calculated to assess the impact of the COVID-19 pandemic on self-perceived overweight. In addition, we investigated which variables influence the vulnerability of the prevalence of self-perceived overweight. We conducted statistical analyses using SAS software (version 9.4; SAS Institute Inc) using a 2-sided test, and statistical significance was defined as a *P* value <.05.

### Ethical Considerations

The KYRBS data were anonymous, and the study protocol was approved by the institutional review board of the Korean Disease Control and Prevention Agency (approval number 2014-06EXP-02-P-A) and the Kyung Hee University and by the local law of the Population Health Promotion Act 19 (approval number 117058) from the Korean government. Every participant provided written informed consent, and the study protocol was performed in accordance with the Declaration of Helsinki.

## Results

**Figure 1:**
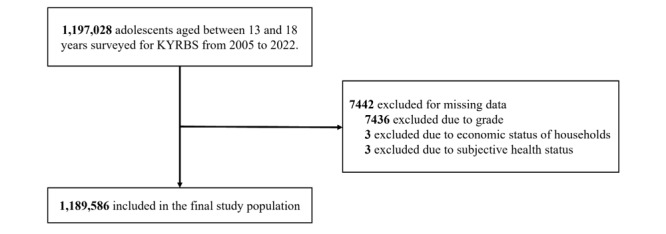
Study workflow. KYRBS, Korea Youth Risk Behavior Web-based Survey.

Table S1 in Multimedia Appendix 1 shows the general characteristics of the participants. From 2005 to 2022, the total number of responses in the KYRBS was 1,197,028. Among these, the number of participants that responded to all questionnaires regarding covariates (except BMI group; categorizing BMI group as unknown if either self-reported weight or height was missing) and self-perceived overweight was 1,189,586, which were selected for analysis in this study. The study workflow of our analysis is illustrated in Figure 1. Of the 1,189,586 respondents, 612,484 (51.49%) were male participants and 577,102 (48.51%) were female participants. There were 615,684 (51.76%) participants in the 7th to 9th grade (middle school) and 573,902 (48.24%) in the 10th to 12th grade (high school). On the basis of their BMI, 89,697 (7.54%) participants were underweight, 836,269 (70.3%) participants were normal, 91,286 (7.67%) participants were overweight, 88,013 (7.4%) participants were obese, and 84,321 (7.09%) participants were unknown.

**Figure 2:**
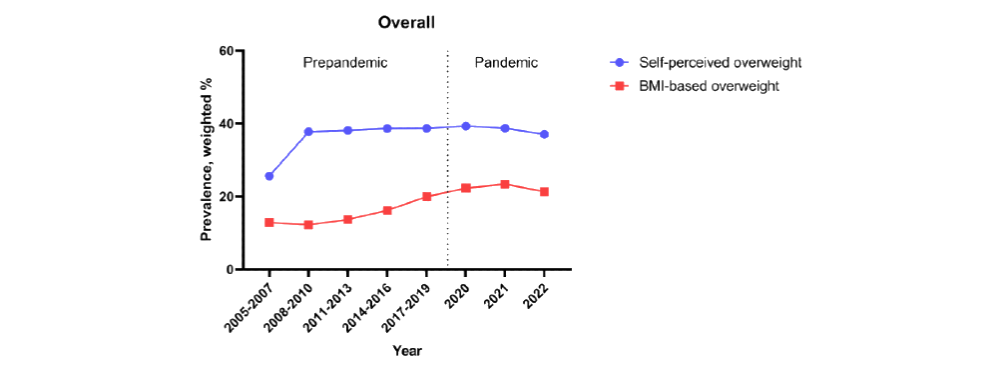
Nationwide trend in self-perceived overweight and BMI-based overweight prevalence over 18 years (2005-2022) among Korean adults (N=1,189,586). BMI was divided into 4 groups following the 2017 Korean National Growth Charts: underweight (<5th percentile), normal (5th-84th percentile), overweight (85th-94th percentile), and obese (≥95th percentile). BMI-based overweight was defined as individuals who were in the overweight or obese groups.

**Figure 3:**
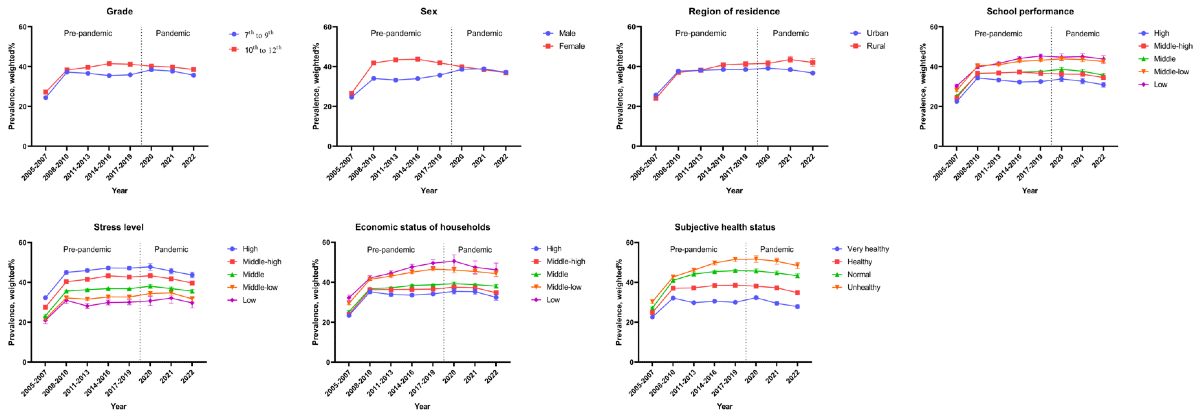
Self-perceived overweight prevalence trends for over 18 years, stratified by grade, sex, region of residence, school performance, stress level, subjective health status, and the economic status of household groups (2005-2022). A high resolution version of this image is available in Multimedia Appendix 2..

Figure 2 indicates that the prevalence of self-perceived overweight is much higher than BMI-based overweight (defined as individuals in the overweight or obese group). Figure 3 and Table S2 in Multimedia Appendix 1 present the self-perceived overweight rate and overweight prevalence from 2005 to 2022, including the COVID-19 prepandemic and pandemic eras. Before the COVID-19 pandemic, the prevalence of self-perceived overweight steadily increased. However, during the pandemic, a decreasing trend was observed. In addition, during the COVID-19 pandemic, groups with higher stress levels or lower economic status of households showed a more significant decrease in self-perceived overweight. From 2005 to 2019 (prepandemic), the overall self-perceived overweight rate increased (β=2.80, 95% CI 2.70-2.90). During 2020 to 2022 (pandemic), the overall self-perceived overweight rate significantly decreased (β=–.53, 95% CI –0.74 to –0.33). During the COVID-19 pandemic, individuals with higher levels of stress showed a greater reduction in the rate of self-perceived overweight as follows: high group (β=–1.20, 95% CI –1.72 to –0.68), middle-high group (β=–1.02, 95% CI –1.36 to –0.68), middle group (β=–.47, 95% CI –0.76 to –0.19), middle-low group (β=–0.19, 95% CI –0.62 to 0.24), low group (β=.10, 95% CI –0.78 to 0.99). Furthermore, during the COVID-19 pandemic, individuals with lower economic status of households presented a more significant decrease in the rate of self-perceived overweight as follows: high group (β=–0.54, 95% CI –1.07 to –0.01), middle-high group (β=–0.58, 95% CI –0.90 to –0.26), middle group (β=–0.23, 95% CI –0.50 to –0.04), middle-low group (β=–0.72, 95% CI –1.28 to –0.15), and low group (β=–1.26, 95% CI –2.45 to –0.07).

Table S3 in Multimedia Appendix 1 shows the wPRs of socioeconomic factors for each year group. Consequently, there was an increase in self-perceived overweight before COVID-19 and a decrease during the pandemic. This includes comparisons between 2005-2007, 2008-2010, 2011-2013, 2014-2016, 2017-2019, 2020, 2021, and 2022. During the COVID-19 pandemic, a decreasing trend is observed in the self-perceived overweight ratio as follows: 2020 (wPR 1.01, 95% CI 0.93-1.09; reference years 2017-2019), 2021 (wPR 0.99, 95% CI 0.88-1.11; reference year 2020), and 2022 (wPR 0.97, 95% CI 0.85-1.12; reference year 2021).

Table 1 illustrates the wPRs of self-perceived overweight according to risk factors. The analysis revealed that poorer values in the examined factors were associated with higher levels of self-perceived overweight. When school performance was low, the self-perceived overweight rate increased as follows: middle-high group (wPR 1.17, 95% CI 1.12-1.22; reference high group), middle group (wPR 1.36, 95% CI 1.31-1.42), middle-low group (wPR 1.59, 95% CI 1.54-1.65), low group (wPR 1.86, 95% CI 1.80-1.93). In addition, as stress levels increase, the proportion of self-perceived overweight individuals rises as follows: middle-low group (wPR 1.25, 95% CI 1.22-1.28; reference low group), middle group (wPR 1.56, 95% CI 1.51-1.61), middle-high group (wPR 1.95, 95% CI 1.87-2.03), and high group (wPR 2.44, 95% CI 2.32-2.56). As subjective health status worsens, the prevalence of self-perceived overweight increases as follows: healthy group (wPR 1.26, 95% CI 1.21-1.31; reference very healthy group), normal group (wPR 1.58, 95% CI 1.53-1.64), and unhealthy group (wPR 1.99, 95% CI 1.93-2.06). The proportion of self-perceived overweight individuals increases with a decrease in the economic status of households as follows: middle-high group (wPR 1.17, 95% CI 1.10-1.25; reference high group), middle group (wPR 1.38, 95% CI 1.30-1.46), middle-low group (wPR 1.62, 95% CI 1.53-1.71), and low group (wPR 1.90, 95% CI 1.80-2.00).

**Table 1:** Prevalence ratios for association between self-perceived overweight prevalence of adolescents and each socioeconomic factor (2005-2022).

		Overall (2005-2022)		Prepandemic (2005-2019)			During the pandemic (2020-2022)	
		wPR^a^ (95% CI)	*P* value	wPR (95% CI)	*P* value	wPR (95% CI)		*P* value
**Grade**								
	7th-9th grade (middle school; reference)	1.00 (reference)	—^b^	1.00 (reference)	—	1.00 (reference)		—
	10th-12th grade (high school)^c^	1.06 (1.05-1.08)	<.001	1.07 (1.05-1.08)	<.001	1.04 (1.01-1.06)		.003
**Sex**								
	Male (reference)	1.00 (reference)	—	1.00 (reference)	—	1.00 (reference)		—
	Female	1.10 (1.09-1.12)^c^	<.001^c^	1.12 (1.11-1.13)^c^	<.001^c^	1.00 (0.98-1.03)		.77
**Region of residence**								
	Urban (reference)	1.00 (reference)	—	1.00 (reference)	—	1.00 (reference)		—
	Rural	1.01 (0.98-1.03)	.66	1.00 (0.97-1.02)	.84	1.07 (1.03-1.12)^c^		<.001^c^
**BMI group^d^**								
	Normal (reference)	1.00 (reference)	—	1.00 (reference)	—	1.00 (reference)		—
	Underweight^c^	0.04 (0.03-0.04)	<.001	0.03 (0.03-0.04)	<.001	0.12 (0.10-0.16)		<.001
	Overweight^c^	25.53 (20.70-31.49)	<.001	28.77 (22.21-37.26)	<.001	8.10 (5.43- 12.07)		<.001
	Obese^c^	651.81 (502.39-845.65)	<.001	827.80 (600.62-1140.91)	<.001	65.55 (39.94-107.56)		<.001
**School performance^e^**								
	High (reference)	1.00 (reference)	—	1.00 (reference)	—	1.00 (reference)		—
	Middle-high^c^	1.17 (1.12-1.22)	<.001	1.16 (1.11-1.22)	<.001	1.22 (1.07-1.39)		.003
	Middle^c^	1.36 (1.31-1.42)	<.001	1.35 (1.30-1.41)	<.001	1.48 (1.32-1.67)		<.001
	Middle-low^c^	1.59 (1.54-1.65)	<.001	1.57 (1.51-1.63)	<.001	1.80 (1.62-2.01)		<.001
	Low^c^	1.86 (1.80-1.93)	<.001	1.83 (1.76-1.89)	<.001	2.20 (1.98-2.44)		<.001
**Stress level^e^**								
	Low (reference)	1.00 (reference)	—	1.00 (reference)	—	1.00 (reference)		—
	Middle-low^c^	1.25 (1.22-1.28)	<.001	1.25 (1.22-1.28)	<.001	1.27 (1.20-1.34)		<.001
	Middle^c^	1.56 (1.51-1.61)	<.001	1.56 (1.51-1.61)	<.001	1.60 (1.48-1.74)		<.001
	Middle-high^c^	1.95 (1.87-2.03)	<.001	1.95 (1.87-2.03)	<.001	2.03 (1.83-2.25)		<.001
	High^c^	2.44 (2.32-2.56)	<.001	2.43 (2.31-2.57)	<.001	2.57 (2.26-2.92)		<.001
**Subjective health status**								
	Very healthy (reference)	1.00 (reference)	—	1.00 (reference)	—	1.00 (reference)		—
	Healthy^c^	1.26 (1.21-1.31)	<.001	1.24 (1.19-1.29)	<.001	1.40 (1.27-1.55)		<.001
	Normal^c^	1.58 (1.53-1.64)	<.001	1.53 (1.47-1.58)	<.001	1.96 (1.79-2.14)		<.001
	Unhealthy^c^	1.99 (1.93-2.06)	<.001	1.89 (1.83-1.95)	<.001	2.74 (2.53-2.98)		<.001
**Economic status of households^e^**								
	High (reference)	1.00 (reference)	—	1.00 (reference)	—	1.00 (reference)		—
	Middle-high	1.17 (1.10-1.25)^c^	<.001^c^	1.17 (1.09-1.26)^c^	<.001^c^	1.26 (0.99-1.62)		.06
	Middle^c^	1.38 (1.30-1.46)	<.001	1.38 (1.29-1.47)	<.001	1.60 (1.28-2.00)		<.001
	Middle-low^c^	1.62 (1.53-1.71)	<.001	1.61 (1.52-1.71)	<.001	2.02 (1.64-2.48)		<.001
	Low^c^	1.90 (1.80-2.00)	<.001	1.89 (1.79-2.00)	<.001	2.55 (2.09-3.10)		<.001

^a^wPR: weighted prevalence ratio.

^b^Not available.

^c^Significant difference (*P*<.05).

^d^BMI was divided into 4 groups according to the 2017 Korean National Growth Charts: underweight (0th-4th percentile), normal (5th-84th percentile), overweight (85th-94th percentile), and obese (95th-100th percentile).

^e^School performance, stress level, and economic status of households were divided into 5 groups: low (0th-19th percentile), middle-low (20th-39th percentile), middle (40th-59th percentile), middle-high (60th-79th percentile), and high (80th-100th percentile).

## Discussion

### Principal Findings

Our study investigated self-perceived overweight among Korean adolescents from 2005 to 2022, spanning 18 years. On the basis of an extensive period and a large population, we examined the difference in the growth rate of self-perceived overweight before and during the COVID-19 pandemic, focusing on risk factors. The prevalence of self-perceived overweight is significantly higher than that of BMI-based overweight. This indicates that individuals who are not overweight based on their BMI consider themselves to be overweight. From 2005 to 2022, the overall prevalence of self-perceived overweight increased from 2005 to 2019 (prepandemic) but decreased from 2020 to 2022 (pandemic). These findings suggest an influence of the COVID-19 pandemic on the rate of self-perceived overweight among adolescents. Furthermore, the rate of self-perceived overweight tends to be higher among individuals with poor academic performance, lower economic status, poorer subjective health, and a higher stress level. This result implies that lower school performance, subjective health status, the economic status of households, and a higher stress level may act as risk factors for self-perceived overweight. In addition, during the COVID-19 pandemic, the rate of self-perceived overweight decreased more significantly among individuals with higher stress levels or lower economic status of households. This finding indicates which demographic groups were more significantly associated with specific factors during the COVID-19 pandemic.

### Plausible Underlying Mechanisms

The prevalence of self-perceived overweight is considerably greater than that of BMI-based overweight. This outcome could be attributed to the fact that individuals who are self-perceived overweight, not BMI-based overweight, frequently engage in comparisons with others who possess more idealized body shapes through media and social interactions [23]. This could lead to the misconception that they perceive themselves as overweight. In addition, this phenomenon might be attributed to normal weight dysmorphia, which is common during adolescence. Normal weight dysmorphia refers to excessive concern or anxiety about one’s physical appearance that often occurs in this age group [24]. Therefore, individuals may perceive themselves as overweight, regardless of their actual weight.

The observed decrease in the prevalence of self-perceived overweight may be primarily attributed to the diminished social activities during the COVID-19 pandemic, which led to a significant reduction in overall social interactions [25]. This decline in social interactions may have consequently led to decreased interdependence among individuals. Participants, because of this diminished interdependence, may have experienced lower stress levels concerning other perceptions and found themselves in fewer situations of self-comparison with others [26]. Because comparing oneself with others is a contributing factor to self-perceived overweight, the reduction in such comparative behaviors may have led to a decrease in self-perceived overweight [27]. However, according to previous studies, various factors, such as increased Social Networking Service use during the COVID-19 pandemic, could also increase the prevalence of self-perceived overweight [28]. Therefore, further studies suggesting more detailed associations between factors that differed during COVID-19, such as lifestyle habits, SNS use, outdoor activities, and the prevalence of self-perceived overweight, are needed. In our study, we found that individuals with lower school performance, lower subjective health status, lower economic status of households, and higher stress levels had a higher prevalence of self-perceived overweight. A potential reason for this phenomenon is that the risk factors diminished the self-esteem of individuals, and this lowered self-esteem, in turn, led to an increase in the prevalence of self-perceived overweight. Lower school performance could lead to frustration, which possibly supports the evidence that it can lead to negative self-esteem [29]. Individuals exhibiting lower subjective health status may experience a loss of self-confidence due to this condition, leading to a decrease in self-esteem. In addition, in previous studies, it has been described that households with lower economic status may also experience lower self-esteem [30]. High stress levels arising from factors, such as maltreatment and parental illness can lead to a reduction in self-esteem [31]. Furthermore, it is observed that individuals with lower self-esteem tend to have a negative perception of their body image [32]. Consequently, the risk factors contribute to a decrease in self-esteem, which may possibly support the evidence of an increase in the prevalence of self-perceived overweight.

As stress levels increased and the economic status of households declined, self-esteem tended to be lower [30,31]. Individuals with lower self-esteem are more prone to making physical appearance comparisons [33]. As previously mentioned, during the COVID-19 period, a decrease in such comparisons coincided with a decrease in the rate of self-perceived overweight. Therefore, individuals with higher stress levels and those with lower economic status of households experienced a more pronounced decrease in self-perceived overweight during the pandemic, likely due to a significant reduction in comparisons.

We can also propose a hypothesis considering the relationship between stress levels and academic performance in the context of self-criticism syndrome. Individuals with self-criticism syndrome engage in constant harsh self-scrutiny and evaluation [34]. Therefore, they are more likely to rate their stress levels and academic performance lower. A previous study also suggested that such self-criticism is associated with the overevaluation of shape and weight independently of self-esteem and depression [35]. Consequently, groups that rate their stress levels and academic performance lower due to self-criticism syndrome may also be more likely to perceive themselves as overweight.

### Comparison With Previous Studies

While a previous study, contrary to our study, has suggested that during the COVID-19 pandemic, rising use of social media has led to an increase in body dissatisfaction among adolescents and young women [28], it did not directly investigate body dissatisfaction year by year; rather, they conducted a single survey during the pandemic to investigate body dissatisfaction based on SNS use before and during the pandemic without considering other risk factors. Our study, in contrast, used the results from an annual survey conducted since 2005 and investigated how body dissatisfaction prevalence changes in relation to various risk factors. Because the survey was conducted before the pandemic, there was no bias based on the survey questions. In addition, due to the diverse range of survey questions, we were able to observe directly how the prevalence of body dissatisfaction changed according to various risk factors.

Several studies have documented the associations between school performance, subjective health status, economic status, stress level, and self-esteem [29-31]. Furthermore, a previous study has indicated that low self-esteem is associated with an increased rate of self-perceived overweight [32]. However, our study is distinct in simultaneously comparing various factors and directly examining their correlation with self-perceived overweight. Our study identified the risk factors associated with self-perceived overweight by referencing previous studies, presenting a novel perspective on the reasons behind the impact of these risk factors. Furthermore, while this study focuses on South Korea, previous studies have been conducted in other countries, potentially leading to varied outcomes due to cultural differences. For instance, as Western and Eastern cultures have different body image and beauty standards, the prevalence of self-perceived overweight could present different trends by country [36]. In addition, disparities in COVID-19 regulations, academic environments, and sociocultural contexts between countries may have influenced the risk factors and the prevalence of self-perceived overweight differently [37,38]. However, as globalization progresses, it may also be considered that the differences between the East and the West are diminishing [39]. This underscores the potential impact of country-specific factors on the results. Therefore, our study has broadened the overall understanding of self-perceived overweigh and laid the foundation for future related studies.

### Clinical and Policy Implications

The number of adolescents experiencing self-perceived overweight is increasing, and experiencing body dissatisfaction during adolescence is likely to continue into adulthood [8]. Furthermore, adolescents who are self-perceived overweight are susceptible to rigorous dieting, body dissatisfaction, and eating disorders, as well as mental health issues such as depression [37,38]. Therefore, it is crucial to address these issues during adolescence. Despite the existing limitations in studies on self-perceived overweight, our study contributes by identifying potential risk factors and elucidating the psychological mechanisms that may underlie self-perceived overweight. Therefore, our findings are likely to facilitate subsequent in-depth study into the psychological issues associated with self-perceived overweight and the factors influencing them. In addition, there is a lack of policies and institutions dedicated to resolving body dissatisfaction among adolescents. Although the number of youth counseling centers has increased, very few specialize in counseling for adolescent body dissatisfaction. In addition, due to the nature of body dissatisfaction, it is often difficult for individuals to recognize the problem themselves, so it is necessary to provide information and education on this topic. However, education in this regard is also insufficient. Therefore, it is necessary to increase the number of counseling centers specializing in adolescent body dissatisfaction and to introduce educational programs that help individuals recognize and address their body dissatisfaction. Establishing such centers and developing programs involve significant capital and time investments. Despite these challenges, these initiatives are meaningful as they have the potential to enhance the mental and physical health of adolescent students. In addition, school health policies should be prepared to form healthy eating habits and to practice appropriate physical activities [40]. Our study indicates that the prevalence of self-perceived overweight decreased during the COVID-19 pandemic and increased due to poor risk factors. Therefore, it is necessary to give adolescents with poor risk factors opportunities to receive mental health care and information. Furthermore, counseling and educational programs designed to assist adolescents with high stress levels and low economic status of households to do less comparison with others and have better social interactions should be provided. This policy requires increased attention from school teachers and periodic surveys to identify students with poor risk factors. However, it offers the utility of effectively improving the mental and physical health of at-risk adolescents through evidence-based interventions. Consequently, collaborative efforts between the Ministry of Health and Welfare and the Ministry of Education are imperative to safeguard the adolescents who are vulnerable [40]. Ultimately, these efforts can serve as specific public health strategies aimed at encouraging a positive body image and preventing negative outcomes.

### Strengths and Limitations

In our study, we used extensive data from over a million Korean adolescents, spanning a substantial period both before and during the COVID-19 pandemic. This extended timeframe allowed us to clearly examine the impact of the pandemic on the self-perceptions of Korean adolescents. Moreover, by incorporating several variables, such as grade, sex, region of residence, BMI, school performance, stress level, subjective health status, smoking status, alcohol consumption, and the economic status of households, we were able to identify potential risk factors influencing the prevalence of self-perceived overweight. Therefore, this study, being the first to use a large sample size, an extended study period, and a comprehensive analysis of various risk factors, has significantly expanded our understanding of self-perceived overweight. In addition, the findings of this study may be beneficial in developing specific public health strategies to encourage a positive body image and prevent negative outcomes. On the basis of our study findings, understanding the factors that influence self-perceived overweight among adolescents enables policy makers and public health professionals to design targeted interventions that promote healthier and more positive body images. Despite these advantages, there are still several limitations to our study. First, in the KYRBS dataset, surveys were conducted in 800 schools, including both middle and high schools. However, according to the Korean Statistical Information Service, as of 2022, there are 3258 middle schools and 2373 high schools. Consequently, the sample may not completely represent all adolescents across every school, and the surveys were limited to students currently enrolled in schools. Despite these constraints, the used data provide comprehensive information on adolescents, and the percentage of students not attending school in South Korea is only 1.8% [10], making it a minor concern. Furthermore, in this study, meticulous and sophisticated sampling methods were used to ensure the accurate representation of as many adolescents as possible.

Second, the nature of surveys relies on the anonymity and voluntary participation of respondents, which could lead to potential issues, such as dishonest responses, underresponse, or overresponse. These circumstances may raise concerns about the reliability of the data. However, the credibility of self-reported surveys, including the KYRBS data, has already been validated through various other studies [41]. Therefore, it is anticipated that such issues will not significantly affect the outcomes.

Third, there appears to be a need for additional questioning regarding the relationship between self-perceived overweight and self-esteem. While we successfully described a correlation between self-perceived overweight, self-esteem, and the impact of risk factors, it was impossible to directly derive a causal relationship based solely on the KYRBS data. Nevertheless, the significance of our study lies in its provision of a valuable direction for future researchers on the phenomenon of self-perceived overweight.

Fourth, the data analysis method we used has inherent limitations that may lead to potential bias. This method can produce biased results if the input data itself are inherently biased. However, the KYRBS data used in our study use stratified cluster sampling, which minimizes bias by considering factors such as school size, grade level, and the type of school. Nonetheless, caution is necessary, as the data analysis approach can still introduce potential biases, particularly if it includes unnecessary variables or if the variables have excessively high correlations.

### Conclusions

Our study, which analyzed the prevalence of self-perceived overweight among 1,189,586 adolescents who participated in KYRBS from 2005 to 2022, revealed that the prevalence of self-perceived overweight is significantly higher than that of BMI-based overweight. In addition, during the COVID-19 pandemic, the prevalence of self-perceived overweight decreased. We also found that groups with lower school performance, subjective health status, and household economic status, as well as those with higher stress levels, were more likely to have a higher self-perceived overweight ratio. The prevalence of self-perceived overweight decreased more significantly among those with higher stress levels or lower economic status of households during the COVID-19 pandemic. These findings show which factors affect self-perceived overweight and further provide information on who is vulnerable to being self-perceived overweight. Our findings suggest policies to prevent diseases arising from self-perceived overweight, particularly for adolescents with poor risk factors. This study indicates the need for further research on factors influencing self-perceived overweight and the impact of COVID-19 on self-perceived overweight.

## Appendix

Multimedia Appendix 1 Baseline characteristics of participants in Korea Youth Risk Behavior Web-based Survey (KYRBS), 2005-2022 (total n=1,189,586). The nationwide trend of self-perceived overweight prevalence before and during the COVID-19 pandemic, weighted % (95% CI), in the KYRBS. Prevalence ratios of self-perceived overweight in socioeconomic factors for each year group participants: 2005-2007, 2008-2010, 2011-2013, 2014-2016, 2017-2019, 2020, 2021, and 2022.

Multimedia Appendix 2 High Resolution Figure 3.

## Abbreviations

KYRBS: Korea Youth Risk Behavior Web-based Survey
wPR: weighted prevalence ratio

## Data Availability

The data are available upon request. Study protocol and statistical code are available from DKY. The dataset is available from the Korea Disease Control and Prevention Agency through a data use agreement.
