# Supplementary material for: National Trends in the Prevalence of Self-Perceived Overweight Among Adolescents Between 2005 and 2022: Nationwide Representative Study
Source: JMIR Public Health Surveill. 2024 Oct 9;10:e57803. doi: 10.2196/57803 (PMC11499719; doi:10.2196/57803)
Supplement: Multimedia Appendix 1 [file publichealth_v10i1e57803_app1.docx]

| **Multimedia Appendix 1**  **Supplementary tables** |
| --- |

**Table S1**. Baseline characteristics of participants in KYRBS, 2005-2022 (total n=1,189,586).

|  | Total | Pre-pandemic | | | | | During the pandemic | | |
| --- | --- | --- | --- | --- | --- | --- | --- | --- | --- |
|  | 2005-2022 | 2005-2007 | 2008-2010 | 2011-2013 | 2014-2016 | 2017-2019 | 2020 | 2021 | 2022 |
| Overall, n | 1,189,586 | 202,748 | 221,266 | 221,102 | 204,521 | 178,664 | 54,809 | 54,712 | 51,764 |
| **Crude value, n (%)** | | | | | | | | | |
| Grade, n (%) | | | | | | | | | |
| 7^th^-9^th^ grade (middle school) | 615,684 (51.76) | 110,795 (54.65) | 113,909 (51.48) | 111,760 (50.55) | 102,204 (49.97) | 90,123 (50.44) | 28,928 (52.78) | 29,980 (54.80) | 27,985 (54.06) |
| 10^th^-12^th^ grade (high school) | 573,902(48.24) | 91,953 (45.35) | 107,357 (48.52) | 109,342 (49.45) | 102,317 (50.03) | 88,541 (49.56) | 25,881 (47.22) | 24,732 (45.20) | 23,779 (45.94) |
| Sex, n (%) | | | | | | | | | |
| Male | 612,484 (51.49) | 105,551 (52.06) | 115,870 (52.37) | 112,039 (50.67) | 104,776 (51.23) | 91,309 (51.11) | 28,269 (51.58) | 28,316 (51.75) | 26,354 (50.91) |
| Female | 577,102 (48.51) | 97,197 (47.94) | 105,396 (47.63) | 109,063 (49.33) | 99,745 (48.77) | 87,355 (48.89) | 26,540 (48.42) | 26,396 (48.25) | 25,410 (49.09) |
| Region of residence, n (%) | | | | | | | | | |
| Urban | 1,061,888 (89.27) | 173,197 (85.42) | 192,484 (86.99) | 194,074 (87.78) | 188,236 (92.04) | 164,864 (92.28) | 50,477 (92.10) | 50,605 (92.49) | 47,951 (92.63) |
| Rural | 127,698 (10.73) | 29,551 (14.58) | 28,782 (13.01) | 27,028 (12.22) | 16,285 (7.96) | 13,800 (7.72) | 4,332 (7.90) | 4,107 (7.51) | 3,813 (7.37) |
| BMI group, n (%) ^a^ | | | | | | | | | |
| Underweight | 89,697 (7.54) | 12,355 (6.09) | 19,843 (8.97) | 17,580 (7.95) | 15,251 (7.46) | 11,969 (6.70) | 4,029 (7.35) | 4,314 (7.88) | 4,356 (8.42) |
| Normal | 836,269 (70.30) | 110,747 (54.62) | 169,168 (76.45) | 168,421 (76.17) | 151,788 (74.22) | 127,399 (71.31) | 37,295 (68.05) | 36,431 (66.59) | 35,020 (67.65) |
| Overweight | 91,286 (7.67) | 10,484 (5.17) | 15,207 (6.87) | 16,642 (7.53) | 16,987 (8.31) | 16,318 (9.13) | 5,531 (10.09) | 5,372 (9.82) | 4,745 (9.17) |
| Obese | 88,013 (7.40) | 8,035 (3.96) | 11,693 (5.28) | 13,414 (6.07) | 15,742 (7.70) | 18,790 (10.52) | 6,679 (12.19) | 7,328 (13.39) | 6,332 (12.23) |
| Unknown | 84,321 (7.09) | 61,127 (30.15) | 5,355 (2.42) | 5,045 (2.28) | 4,753 (2.32) | 4,188 (2.34) | 1,275 (2.33) | 1,267 (2.32) | 1,311 (2.53) |
| School performance, n (%) ^b^ | | | | | | | | | |
| High | 145,866 (12.26) | 26,755 (13.20) | 25,004 (11.30) | 23,878 (10.80) | 25,733 (12.58) | 23,847 (13.35) | 6,699 (12.22) | 7,036 (12.86) | 6,914 (13.36) |
| Middle-high | 299,662 (25.19) | 58,032 (28.62) | 52,108 (23.55) | 52,753 (23.86) | 51,347 (25.11) | 45,504 (25.47) | 13,386 (24.42) | 13,426 (24.54) | 13,106 (25.32) |
| Middle | 339,055 (28.50) | 59,998 (29.59) | 59,983 (27.11) | 60,165 (27.21) | 57,586 (28.16) | 52,432 (29.35) | 16,555 (30.20) | 16,873 (30.84) | 15,463 (29.87) |
| Middle-low | 278,462 (23.41) | 41,817 (20.63) | 56,700 (25.63) | 56,167 (25.40) | 48,212 (23.57) | 39,544 (22.13) | 12,665 (23.11) | 11,990 (21.91) | 11,367 (21.96) |
| Low | 126,541 (10.64) | 16,146 (7.96) | 27,471 (12.42) | 28,139 (12.73) | 21,643 (10.58) | 17,337 (9.70) | 5,504 (10.04) | 5,387 (9.85) | 4,914 (9.49) |
| Stress level, n (%) ^b^ | | | | | | | | | |
| High | 138,916 (11.68) | 28,008 (13.81) | 28,680 (12.96) | 26,144 (11.82) | 19,284 (9.43) | 19,946 (11.16) | 4,572 (8.34) | 5,963 (10.90) | 6,319 (12.21) |
| Middle-high | 350,148 (29.43) | 65,601 (32.36) | 67,988 (30.73) | 67,033 (30.32) | 55,275 (27.03) | 49,971 (27.97) | 14,023 (25.59) | 15,223 (27.82) | 15,034 (29.04) |
| Middle | 492,891 (41.43) | 79,577 (39.25) | 90,216 (40.77) | 91,386 (41.33) | 88,541 (43.29) | 74,046 (41.44) | 24,335 (44.40) | 23,175 (42.36) | 21,615 (41.76) |
| Middle-low | 174,019 (14.63) | 25,557 (12.61) | 29,670 (13.41) | 30,950 (14.00) | 34,084 (16.67) | 28,014 (15.68) | 9,870 (18.01) | 8,568 (15.66) | 7,306 (14.11) |
| Low | 33,612 (2.83) | 4,005 (1.98) | 4,712 (2.13) | 5,589 (2.53) | 7,337 (3.59) | 6,687 (3.74) | 2,009 (3.67) | 1,783 (3.26) | 1,490 (2.88) |
| Subjective health status, n (%) | | | | | | | | | |
| Very healthy | 255,820 (21.51) | 33,447 (16.50) | 38,764 (17.52) | 43,647 (19.74) | 52,658 (25.75) | 49,548 (27.73) | 15,097 (27.54) | 12,139 (22.19) | 10,520 (20.32) |
| Healthy | 540,587 (45.44) | 91,172 (44.97) | 102,334 (46.25) | 105,438 (47.69) | 94,491 (46.20) | 78,275 (43.81) | 23,258 (42.43) | 23,308 (42.60) | 22,311 (43.10) |
| Normal | 302,035 (25.39) | 59,463 (29.33) | 62,131 (28.08) | 56,426 (25.52) | 45,048 (22.03) | 38,852 (21.75) | 12,309 (22.46) | 14,262 (26.07) | 13,544 (26.16) |
| Unhealthy | 91,144 (7.66) | 18,666 (9.21) | 18,037 (8.15) | 15,591 (7.05) | 12,324 (6.03) | 11,989 (6.71) | 4,145 (7.56) | 5,003 (9.14) | 5,389 (10.41) |
| Smoking status, n (%) | | | | | | | | | |
| Non-smoker | 944,919 (79.43) | 145,295 (71.66) | 160,310 (72.45) | 168,146 (76.05) | 169,962 (83.10) | 155,316 (86.93) | 49,221 (89.80) | 49,429 (90.34) | 47,240 (91.26) |
| Smoker | 244,667 (20.57) | 57,453 (28.34) | 60,956 (27.55) | 52,956 (23.95) | 34,559 (16.90) | 23,348 (13.07) | 5,588 (10.20) | 5,283 (9.66) | 4,524 (8.74) |
| Alcohol consumption, n (%) | | | | | | | | | |
| 0 days/month | 965,155 (81.13) | 146,077 (72.05) | 171,883 (77.68) | 179,771 (81.31) | 172,957 (84.57) | 151,420 (84.75) | 48,974 (89.35) | 48,951 (89.47) | 45,122 (87.17) |
| 1–5 days/month | 169,966 (14.29) | 41,675 (20.56) | 36,034 (16.29) | 31,580 (14.28) | 24,956 (12.20) | 21,401 (11.98) | 4,525 (8.26) | 4,520 (8.26) | 5,275 (10.19) |
| 6–30 days/month | 54,465 (4.58) | 14,996 (7.40) | 13,349 (6.03) | 9,751 (4.41) | 6,608 (3.23) | 5,843 (3.27) | 1,310 (2.39) | 1,241 (2.27) | 1,367 (2.64) |
| Economic status of households, n (%) ^b^ | | | | | | | | | |
| High | 96,574 (8.12) | 13,934 (6.87) | 13,311 (6.02) | 14,468 (6.54) | 17,729 (8.67) | 19,270 (10.79) | 6,000 (10.95) | 5,906 (10.79) | 5,956 (11.51) |
| Middle-high | 313,440 (26.35) | 59,921 (29.55) | 48,019 (21.70) | 52,468 (23.73) | 54,491 (26.64) | 51,779 (28.98) | 15,271 (27.86) | 15,602 (28.52) | 15,889 (30.70) |
| Middle | 558,662 (46.96) | 90,056 (44.42) | 104,752 (47.34) | 104,881 (47.44) | 97,831 (47.83) | 83,624 (46.81) | 26,357 (48.09) | 27,037 (49.42) | 24,124 (46.60) |
| Middle-low | 173,135 (14.55) | 30,277 (14.93) | 40,974 (18.52) | 38,405 (17.37) | 27,854 (13.62) | 19,831 (11.10) | 5,919 (10.80) | 5,074 (9.27) | 4,801 (9.27) |
| Low | 47,775 (4.02) | 8,560 (4.22) | 14,210 (6.42) | 10,880 (4.92) | 6,616 (3.23) | 4,160 (2.33) | 1,262 (2.30) | 1,093 (2.00) | 994 (1.92) |
| **Weighted % (95% CI)** | | | | | | | | | |
| Grade, weighted % (95% CI) | | | | | | | | | |
| 7^th^-9^th^ grade (middle school) | 50.35 (49.97 to 50.73) | 56.22 (55.32 to 57.12) | 50.85 (49.93 to 51.77) | 49.14 (48.35 to 49.93) | 46.98 (46.18 to 47.77) | 46.53 (45.69 to 47.37) | 49.67 (48.19 to 51.14) | 51.04 (49.61 to 52.47) | 51.68 (50.13 to 53.22) |
| 10^th^-12^th^ grade (high school) | 49.65 (49.27 to 50.03) | 43.78 (42.88 to 44.68) | 49.15 (48.23 to 50.07) | 50.86 (50.07 to 51.65) | 53.02 (52.23 to 53.82) | 53.47 (52.63 to 54.31) | 50.33 (48.86 to 51.81) | 48.96 (47.53 to 50.39) | 48.32 (46.78 to 49.87) |
| Sex, weighted % (95% CI) | | | | | | | | | |
| Male | 52.25 (51.64 to 52.86) | 53.00 (51.44 to 54.55) | 52.84 (51.25 to 54.42) | 52.44 (51.02 to 53.87) | 52.09 (50.64 to 53.54) | 51.96 (50.55 to 53.38) | 51.84 (49.57 to 54.10) | 51.64 (49.46 to 53.82) | 51.56 (49.40 to 53.72) |
| Female | 47.75 (47.14 to 48.36) | 47.00 (45.45 to 48.56) | 47.16 (45.58 to 48.75) | 47.56 (46.13 to 48.98) | 47.91 (46.46 to 49.36) | 48.04 (46.62 to 49.45) | 48.16 (45.90 to 50.43) | 48.36 (46.18 to 50.54) | 48.44 (46.28 to 50.60) |
| Region of residence, weighted % (95% CI) | | | | | | | | | |
| Urban | 93.85 (93.63 to 94.07) | 92.54 (91.95 to 93.13) | 94.29 (93.89 to 94.68) | 93.55 (93.02 to 94.08) | 93.77 (93.22 to 94.33) | 94.05 (93.47 to 94.64) | 94.11 (93.32 to 94.90) | 94.45 (93.67 to 95.24) | 94.42 (93.51 to 95.33) |
| Rural | 6.15 (5.93 to 6.37) | 7.46 (6.87 to 8.05) | 5.72 (5.32 to 6.11) | 6.45 (5.92 to 6.98) | 6.23 (5.67 to 6.78) | 5.95 (5.36 to 6.53) | 5.89 (5.10 to 6.68) | 5.55 (4.76 to 6.33) | 5.58 (4.67 to 6.49) |
| BMI group, weighted % (95% CI) ^a^ | | | | | | | | | |
| Underweight | 7.75 (7.69 to 7.82) | 6.10 (5.95 to 6.25) | 9.04 (8.89 to 9.19) | 8.13 (7.99 to 8.26) | 7.54 (7.41 to 7.68) | 6.81 (6.68 to 6.94) | 7.56 (7.31 to 7.81) | 8.15 (7.89 to 8.40) | 8.68 (8.43 to 8.93) |
| Normal | 69.77 (69.62 to 69.93) | 54.36 (53.67 to 55.05) | 76.62 (76.38 to 76.87) | 76.29 (76.07 to 76.52) | 74.35 (74.11 to 74.60) | 71.44 (71.17 to 71.71) | 68.49 (67.99 to 68.99) | 66.81 (66.29 to 67.33) | 68.15 (67.63 to 68.68) |
| Overweight | 7.94 (7.88 to 8.00) | 5.05 (4.93 to 5.18) | 6.83 (6.70 to 6.96) | 7.39 (7.27 to 7.51) | 8.21 (8.08 to 8.33) | 9.11 (8.98 to 9.25) | 9.95 (9.68 to 10.21) | 9.70 (9.44 to 9.96) | 8.99 (8.72 to 9.26) |
| Obese | 8.22 (8.14 to 8.30) | 3.87 (3.75 to 3.99) | 5.14 (5.01 to 5.28) | 6.00 (5.88 to 6.12) | 7.62 (7.47 to 7.77) | 10.38 (10.19 to 10.56) | 11.86 (11.49 to 12.22) | 13.20 (12.80 to 13.59) | 11.83 (11.45 to 12.22) |
| Unknown | 6.31 (6.17 to 6.45) | 30.62 (29.79 to 31.45) | 2.36 (2.29 to 2.44) | 2.19 (2.12 to 2.26) | 2.28 (2.21 to 2.35) | 2.26 (2.18 to 2.33) | 2.14 (2.01 to 2.28) | 2.15 (2.01 to 2.29) | 2.35 (2.20 to 2.49) |
| School performance, weighted % (95% CI) ^b^ | | | | | | | | | |
| High | 12.37 (12.28 to 12.47) | 13.53 (13.31 to 13.75) | 11.34 (11.14 to 11.55) | 10.81 (10.63 to 10.99) | 12.44 (12.24 to 12.64) | 13.21 (13.00 to 13.42) | 12.19 (11.79 to 12.59) | 12.64 (12.29 to 12.98) | 13.47 (13.05 to 13.89) |
| Middle-high | 25.19 (25.09 to 25.30) | 28.88 (28.62 to 29.13) | 23.67 (23.45 to 23.88) | 23.98 (23.78 to 24.18) | 25.05 (24.84 to 25.25) | 25.37 (25.14 to 25.59) | 24.65 (24.23 to 25.08) | 24.48 (24.05 to 24.92) | 25.35 (24.92 to 25.78) |
| Middle | 28.89 (28.79 to 29.00) | 29.25 (28.98 to 29.51) | 27.17 (26.93 to 27.41) | 27.33 (27.12 to 27.53) | 28.25 (28.03 to 28.47) | 29.46 (29.23 to 29.70) | 30.16 (29.74 to 30.57) | 31.03 (30.61 to 31.44) | 30.03 (29.60 to 30.46) |
| Middle-low | 23.16 (23.05 to 23.27) | 20.44 (20.20 to 20.68) | 25.68 (25.43 to 25.93) | 25.36 (25.15 to 25.58) | 23.65 (23.44 to 23.87) | 22.15 (21.92 to 22.38) | 23.01 (22.55 to 23.47) | 22.01 (21.61 to 22.41) | 21.76 (21.31 to 22.21) |
| Low | 10.38 (10.31 to 10.46) | 7.91 (7.74 to 8.08) | 12.15 (11.97 to 12.32) | 12.52 (12.35 to 12.68) | 10.61 (10.45 to 10.77) | 9.81 (9.65 to 9.97) | 10.00 (9.68 to 10.31) | 9.84 (9.53 to 10.15) | 9.39 (9.08 to 9.70) |
| Stress level, weighted % (95% CI) ^b^ | | | | | | | | | |
| High | 11.40 (11.31 to 11.48) | 13.72 (13.51 to 13.93) | 12.78 (12.58 to 12.98) | 11.59 (11.41 to 11.76) | 9.44 (9.29 to 9.60) | 11.11 (10.91 to 11.30) | 8.25 (7.96 to 8.54) | 10.93 (10.60 to 11.25) | 12.29 (11.95 to 12.63) |
| Middle-high | 29.14 (29.02 to 29.25) | 32.48 (32.20 to 32.76) | 30.77 (30.49 to 31.04) | 30.19 (29.94 to 30.44) | 27.11 (26.85 to 27.36) | 27.98 (27.70 to 28.27) | 25.87 (25.40 to 26.33) | 27.82 (27.37 to 28.27) | 29.03 (28.60 to 29.46) |
| Middle | 41.85 (41.74 to 41.97) | 39.20 (38.92 to 39.48) | 40.97 (40.69 to 41.24) | 41.61 (41.37 to 41.85) | 43.47 (43.24 to 43.71) | 41.72 (41.46 to 41.97) | 44.49 (44.05 to 44.94) | 42.58 (42.12 to 43.03) | 41.89 (41.41 to 42.36) |
| Middle-low | 14.75 (14.66 to 14.84) | 12.60 (12.40 to 12.79) | 13.35 (13.15 to 13.55) | 14.07 (13.89 to 14.26) | 16.46 (16.25 to 16.66) | 15.51 (15.29 to 15.73) | 17.81 (17.40 to 18.23) | 15.48 (15.12 to 15.84) | 13.94 (13.60 to 14.28) |
| Low | 2.87 (2.82 to 2.91) | 2.00 (1.91 to 2.09) | 2.13 (2.04 to 2.22) | 2.54 (2.46 to 2.62) | 3.52 (3.42 to 3.62) | 3.69 (3.58 to 3.80) | 3.58 (3.41 to 3.75) | 3.19 (3.02 to 3.36) | 2.85 (2.69 to 3.01) |
| Subjective health status, weighted % (95% CI) | | | | | | | | | |
| Very healthy | 21.75 (21.62 to 21.88) | 16.82 (16.57 to 17.06) | 17.77 (17.51 to 18.03) | 19.99 (19.72 to 20.26) | 25.57 (25.27 to 25.87) | 27.46 (27.13 to 27.79) | 27.17 (26.56 to 27.77) | 22.09 (21.60 to 22.58) | 20.17 (19.69 to 20.65) |
| Healthy | 44.95 (44.84 to 45.07) | 45.04 (44.75 to 45.33) | 46.46 (46.20 to 46.73) | 47.83 (47.60 to 48.06) | 46.23 (45.99 to 46.48) | 43.75 (43.49 to 44.01) | 42.47 (42.02 to 42.92) | 42.60 (42.14 to 43.06) | 42.98 (42.50 to 43.47) |
| Normal | 25.30 (25.19 to 25.41) | 28.95 (28.69 to 29.21) | 27.73 (27.46 to 28.01) | 25.24 (25.00 to 25.48) | 22.11 (21.88 to 22.34) | 21.95 (21.71 to 22.18) | 22.65 (22.22 to 23.08) | 26.08 (25.61 to 26.54) | 26.30 (25.84 to 26.76) |
| Unhealthy | 7.99 (7.93 to 8.06) | 9.19 (9.02 to 9.37) | 8.03 (7.88 to 8.18) | 6.94 (6.81 to 7.07) | 6.08 (5.96 to 6.21) | 6.85 (6.71 to 6.99) | 7.71 (7.44 to 7.99) | 9.23 (8.96 to 9.51) | 10.54 (10.24 to 10.84) |
| Smoking status, weighted % (95% CI) | | | | | | | | | |
| Non-smoker | 81.55 (81.38 to 81.72) | 72.37 (71.93 to 72.81) | 73.04 (72.59 to 73.50) | 76.07 (75.63 to 76.51) | 82.71 (82.28 to 83.13) | 86.38 (86.03 to 86.73) | 89.84 (89.36 to 90.31) | 90.18 (89.73 to 90.63) | 91.18 (90.74 to 91.61) |
| Smoker | 18.45 (18.28 to 18.62) | 27.63 (27.19 to 28.07) | 26.96 (26.50 to 27.42) | 23.93 (23.49 to 24.37) | 17.29 (16.87 to 17.72) | 13.62 (13.27 to 13.97) | 10.17 (9.69 to 10.64) | 9.82 (9.37 to 10.27) | 8.82 (8.39 to 9.26) |
| Alcohol consumption, weighted % (95% CI) | | | | | | | | | |
| 0 days/month | 82.48 (82.34 to 82.63) | 72.53 (72.12 to 72.93) | 77.96 (77.60 to 78.32) | 81.39 (81.05 to 81.72) | 84.01 (83.70 to 84.32) | 84.16 (83.87 to 84.46) | 89.41 (89.01 to 89.82) | 89.33 (88.91 to 89.76) | 87.01 (86.52 to 87.50) |
| 1–5 days/month | 13.35 (13.24 to 13.46) | 20.31 (20.00 to 20.62) | 16.13 (15.87 to 16.39) | 14.22 (13.98 to 14.47) | 12.64 (12.40 to 12.87) | 12.41 (12.17 to 12.64) | 8.25 (7.93 to 8.57) | 8.38 (8.04 to 8.71) | 10.35 (9.94 to 10.75) |
| 6–30 days/month | 4.17 (4.11 to 4.22) | 7.17 (6.98 to 7.35) | 5.92 (5.74 to 6.09) | 4.39 (4.26 to 4.52) | 3.35 (3.23 to 3.47) | 3.43 (3.31 to 3.55) | 2.34 (2.17 to 2.50) | 2.29 (2.12 to 2.46) | 2.64 (2.46 to 2.82) |
| Economic status of households, weighted % (95% CI) ^b^ | | | | | | | | | |
| High | 8.93 (8.83 to 9.03) | 7.41 (7.22 to 7.60) | 6.34 (6.16 to 6.53) | 6.83 (6.66 to 7.01) | 8.74 (8.54 to 8.94) | 10.90 (10.68 to 11.13) | 11.20 (10.74 to 11.67) | 10.83 (10.44 to 11.21) | 11.85 (11.39 to 12.30) |
| Middle-high | 27.63 (27.48 to 27.78) | 31.04 (30.61 to 31.47) | 22.69 (22.36 to 23.02) | 24.33 (24.03 to 24.63) | 26.86 (26.55 to 27.17) | 29.36 (29.04 to 29.67) | 28.65 (28.10 to 29.20) | 29.32 (28.71 to 29.93) | 31.42 (30.85 to 32.00) |
| Middle | 46.75 (46.59 to 46.90) | 43.69 (43.31 to 44.07) | 47.05 (46.74 to 47.36) | 47.11 (46.83 to 47.39) | 47.60 (47.29 to 47.92) | 46.54 (46.21 to 46.88) | 47.57 (46.92 to 48.22) | 49.00 (48.35 to 49.65) | 46.05 (45.36 to 46.73) |
| Middle-low | 13.20 (13.10 to 13.30) | 14.03 (13.77 to 14.30) | 17.86 (17.60 to 18.13) | 16.96 (16.70 to 17.21) | 13.56 (13.34 to 13.77) | 10.90 (10.70 to 11.10) | 10.39 (10.06 to 10.71) | 8.96 (8.64 to 9.28) | 8.82 (8.50 to 9.14) |
| Low | 3.49 (3.44 to 3.53) | 3.83 (3.70 to 3.95) | 6.05 (5.90 to 6.20) | 4.77 (4.65 to 4.88) | 3.24 (3.14 to 3.33) | 2.30 (2.22 to 2.38) | 2.19 (2.06 to 2.32) | 1.89 (1.77 to 2.01) | 1.86 (1.74 to 1.99) |

Abbreviations: BMI, body mass index; CI, confidence interval; KYRBS, Korea Youth Risk Behavior Web-Based Survey.

^a^ BMI was divided into four groups according to the 2017 Korean National Growth Charts: underweight (0-4 percentile), normal (5-84 percentile), overweight (85-94 percentile), and obese (95-100 percentile).

^b^ School performance, stress level, and economic status of households were divided into five groups: low (0-19 percentile), middle-low (20-39 percentile), middle (40-59 percentile), middle-high (60-79 percentile), and high (80-100 percentile).

**Table S2**. The nationwide trend of self-perceived overweight prevalence before and during the COVID-19 pandemic, weighted % (95% CI), in the Korea Youth Risk Behavior Web–based Survey.

|  | | | | | | | | | | | | | Prepandemic, weighted % (95% CI) | | | | | During the pandemic, weighted % (95% CI) | | | Trends in the prepandemic era, β (95% CI)^a^ | Trends in the pandemic era, β (95% CI)^a^ | Trend differences, β_diff_ (95% CI)^a^ |
| --- | --- | --- | --- | --- | --- | --- | --- | --- | --- | --- | --- | --- | --- | --- | --- | --- | --- | --- | --- | --- | --- | --- | --- |
|  | | | | | | | | | | | | | 2005-2007 | 2008-2010 | 2011-2013 | 2014-2016 | 2017-2019 | 2020 | 2021 | 2022 |  |  |  |
|  |  |  | |  |  |  |  |  |  |  |  |  |  |  |  |  |  |  |  |  |  |  |  |
| Overall | | | | | | | | | | | | | 25.64 (25.36-25.92) | 37.74 (37.44-38.05) | 38.14 (37.85-38.44) | 38.68 (38.36-38.99) | 38.72 (38.41-39.02) | 39.32 (38.79-39.85) | 38.74 (38.18-39.29) | 37.08 (36.53-37.64) | 2.80 (2.70-2.90)^b^ | −0.53 (−0.74 to −0.33)^b^ | −3.33 (−3.56 to −3.10)^b^ |
| **Grade** | | | | | | | | | | | | | | | | | | | | | | | |
|  | | | 7th-9th grade (middle school) | | | | | | | | | | 24.43 (24.09-24.77) | 37.21 (36.84-37.58) | 36.62 (36.27-36.96) | 35.50 (35.12-35.88) | 35.87 (35.49-36.26) | 38.40 (37.70-39.11) | 37.75 (36.99-38.51) | 35.71 (34.96-36.46) | 2.36 (2.23-2.49)^b^ | −0.12 (−0.39 to 0.16) | −2.48 (−2.78 to −2.18)^b^ |
|  | | | 10th-12th grade (high school) | | | | | | | | | | 27.20 (26.74-27.66) | 38.30 (37.80-38.79) | 39.61 (39.14-40.09) | 41.49 (41.03-41.96) | 41.19 (40.75-41.64) | 40.22 (39.44-41.01) | 39.77 (38.96-40.57) | 38.55 (37.75-39.36) | 3.07 (2.93-3.22)^b^ | −0.84 (−1.13 to −0.54)^b^ | −3.91 (−4.24 to −3.58)^b^ |
| **Sex** | | | | | | | | | | | | | | | | | | | | | | | |
|  | | | Male | | | | | | | | | | 24.71 (24.38-25.04) | 34.08 (33.74-34.43) | 33.29 (32.99-33.60) | 34.01 (33.66-34.35) | 35.75 (35.40-36.11) | 38.72 (38.03-39.40) | 38.94 (38.24-39.65) | 37.18 (36.49-37.88) | 2.23 (2.12-2.35)^b^ | 0.49 (0.24-0.74)^b^ | −1.74 (−2.02 to −1.47)^b^ |
|  | | | Female | | | | | | | | | | 26.69 (26.26-27.12) | 41.84 (41.44-42.25) | 43.49 (43.13-43.85) | 43.75 (43.34-44.17) | 41.93 (41.48-42.37) | 39.97 (39.21-40.73) | 38.52 (37.71-39.33) | 36.98 (36.20-37.76) | 3.37 (3.23-3.52)^b^ | −1.63 (−1.92 to −1.34)^b^ | −5.00 (−5.32 to −4.68)^b^ |
| **Region of residence** | | | | | | | | | | | | | | | | | | | | | | | |
|  | | | Urban | | | | | | | | | | 25.77 (25.47-26.07) | 37.78 (37.46-38.10) | 38.14 (37.83-38.45) | 38.53 (38.20-38.86) | 38.55 (38.24-38.87) | 39.18 (38.62-39.73) | 38.46 (37.88-39.03) | 36.79 (36.22-37.36) | 2.70 (2.60-2.81)^b^ | −0.59 (−0.80 to −0.38)^b^ | −3.29 (−3.53 to −3.06)^b^ |
|  | | | Rural | | | | | | | | | | 24.04 (23.38-24.70) | 37.12 (36.38-37.85) | 38.18 (37.27-39.09) | 40.90 (39.79-42.01) | 41.32 (40.12-42.52) | 41.61 (40.13-43.09) | 43.56 (42.00-45.11) | 42.09 (40.19-43.98) | 4.16 (3.85-4.47)^b^ | 0.44 (−0.26 to 1.13) | −3.72 (−4.48 to −2.96)^b^ |
| **BMI group^c^** | | | | | | | | | | | | | | | | | | | | | | | |
|  | | | Underweight | | | | | | | | | | 1.16 (0.90-1.41) | 1.69 (1.46-1.92) | 1.52 (1.32-1.73) | 1.88 (1.64-2.12) | 1.60 (1.35-1.86) | 1.41 (1.03-1.80) | 0.93 (0.64-1.22) | 1.11 (0.78-1.45) | 0.10 (0.02-0.18)^b^ | −0.19 (−0.33 to −0.06)^b^ | −0.10 (−0.13 to −0.08)^b^ |
|  | | | Normal | | | | | | | | | | 16.3-5 (15.97-16.72) | 31.50 (31.14-31.87) | 30.68 (30.33-31.03) | 29.13 (28.77-29.48) | 26.02 (25.66-26.37) | 25.31 (24.75-25.88) | 23.66 (23.05-24.26) | 23.26 (22.71-23.82) | 1.40 (1.27-1.52)^b^ | −0.99 (−1.20 to −0.78)^b^ | −0.34 (−0.56 to −0.13)^b^ |
|  | | | Overweight | | | | | | | | | | 88.39 (87.65-89.12) | 95.41 (94.96-95.86) | 94.55 (94.14-94.95) | 92.68 (92.23-93.13) | 89.49 (88.96-90.02) | 86.43 (85.36-87.50) | 86.22 (85.20-87.23) | 88.21 (87.15-89.27) | −0.33 (−0.52 to −0.15)^b^ | −0.44 (−0.83 to −0.06)^b^ | −1.76 (−1.99 to −1.54)^b^ |
|  | | | Obese | | | | | | | | | | 96.95 (96.11-97.79) | 98.63 (98.37-98.88) | 98.09 (97.82-98.37) | 98.09 (97.86-98.33) | 97.37 (97.10-97.64) | 96.95 (96.51-97.39) | 97.10 (96.67-97.53) | 96.60 (96.06-97.14) | −0.06 (−0.21 to 0.09) | −0.21 (−0.41 to −0.02)^b^ | −1.34 (−1.49 to −1.20)^b^ |
| **School performance^d^** | | | | | | | | | | | | | | | | | | | | | | | |
|  | | | High | | | | | | | | | | 22.54 (21.86-23.21) | 34.37 (33.60-35.15) | 33.36 (32.69-34.04) | 32.34 (31.69-33.00) | 32.55 (31.87-33.22) | 33.81 (32.56-35.07) | 32.74 (31.44-34.04) | 30.92 (29.67-32.18) | 1.97 (1.75-2.19)^b^ | −0.57 (−1.03 to −0.11)^b^ | −1.31 (−1.48 to −1.14)^b^ |
|  | | | Middle-high | | | | | | | | | | 24.44 (24.00-24.88) | 36.74 (36.23-37.26) | 36.88 (36.41-37.36) | 37.28 (36.79-37.77) | 36.68 (36.17-37.19) | 36.29 (35.31-37.28) | 36.22 (35.26-37.18) | 34.60 (33.65-35.55) | 2.76 (2.60-2.92)^b^ | −0.63 (−0.97 to −0.28)^b^ | −1.62 (−2.10 to −1.15)^b^ |
|  | | | Middle | | | | | | | | | | 25.29 (24.82-25.75) | 36.47 (35.98-36.96) | 36.92 (36.46-37.38) | 37.28 (36.81-37.75) | 37.61 (37.13-38.09) | 38.70 (37.83-39.56) | 37.71 (36.87-38.55) | 35.78 (34.92-36.65) | 2.65 (2.50-2.80)^b^ | −0.63 (−0.94 to −0.31)^b^ | −1.09 (−1.27 to −0.91)^b^ |
|  | | | Middle-low | | | | | | | | | | 28.09 (27.50-28.68) | 40.47 (39.93-41.01) | 40.96 (40.45-41.47) | 42.70 (42.14-43.26) | 43.25 (42.67-43.84) | 43.92 (42.93-44.90) | 43.59 (42.51-44.68) | 42.65 (41.58-43.71) | 3.19 (3.00-3.38)^b^ | −0.20 (−0.59 to 0.19) | −1.28 (−1.42 to −1.14)^b^ |
|  | | | Low | | | | | | | | | | 30.33 (29.43-31.23) | 39.92 (39.15-40.69) | 41.63 (40.92-42.35) | 44.15 (43.37-44.94) | 45.39 (44.52-46.27) | 44.79 (43.16-46.43) | 45.11 (43.64-46.58) | 43.88 (42.24-45.52) | 3.29 (3.01-3.56)^b^ | −0.42 (−1.01 to 0.17) | −1.29 (−1.42 to −1.16)^b^ |
| **Stress level^d^** | | | | | | | | | | | | | | | | | | | | | | | |
|  | | | High | | | | | | | | | | 32.29 (31.56-33.02) | 45.03 (44.24-45.82) | 45.96 (45.23-46.69) | 47.26 (46.43-48.09) | 47.15 (46.36-47.95) | 47.79 (46.05-49.53) | 45.68 (44.31-47.06) | 43.73 (42.33-45.13) | 3.44 (3.20-3.69)^b^ | −1.20 (−1.72 to −0.68)^b^ | −2.04 (−2.23 to −1.86)^b^ |
|  | | | Middle-high | | | | | | | | | | 27.54 (27.10-27.99) | 40.34 (39.84-40.84) | 41.52 (41.05-41.99) | 43.32 (42.80-43.84) | 42.66 (42.16-43.16) | 43.36 (42.43-44.29) | 41.84 (40.87-42.81) | 39.70 (38.78-40.61) | 3.54 (3.38-3.70)^b^ | −1.02 (−1.36 to −0.68)^b^ | −2.75 (−3.13 to −2.38)^b^ |
|  | | | Middle | | | | | | | | | | 23.23 (22.82-23.64) | 35.67 (35.27-36.07) | 36.34 (35.97-36.72) | 36.89 (36.50-37.27) | 36.84 (36.42-37.25) | 38.06 (37.33-38.78) | 36.88 (36.14-37.62) | 35.60 (34.82-36.39) | 2.88 (2.75-3.02)^b^ | −0.47 (−0.76 to −0.19)^b^ | −1.57 (−1.78 to −1.37)^b^ |
|  | | | Middle-low | | | | | | | | | | 21.74 (21.09-22.39) | 32.22 (31.57-32.86) | 31.58 (31.00-32.17) | 32.72 (32.15-33.29) | 32.68 (32.07-33.30) | 34.42 (33.35-35.50) | 34.74 (33.55-35.92) | 31.74 (30.53-32.96) | 2.20 (2.00-2.40)^b^ | −0.19 (−0.62 to 0.24) | −1.49 (−1.62 to −1.36)^b^ |
|  | | | Low | | | | | | | | | | 20.92 (19.20-22.63) | 31.03 (29.37-32.68) | 28.14 (26.83-29.44) | 29.91 (28.73-31.10) | 30.07 (28.79-31.34) | 30.73 (28.51-32.96) | 32.12 (29.63-34.60) | 29.70 (27.16-32.23) | 1.57 (1.09-2.04)^b^ | 0.10 (−0.78 to 0.99) | −1.24 (−1.35 to −1.13)^b^ |
| **Subjective health status** | | | | | | | | | | | | | | | | | | | | | | | |
|  | | | Very healthy | | | | | | | | | | 22.60 (21.99-23.21) | 32.15 (31.54-32.77) | 29.84 (29.33-30.34) | 30.57 (30.10-31.04) | 30.00 (29.51-30.48) | 32.33 (31.50-33.16) | 29.51 (28.62-30.41) | 27.82 (26.90-28.74) | 1.19 (1.01-1.36)^b^ | −0.83 (−1.16 to −0.50)^b^ | −1.46 (−1.63 to −1.29)^b^ |
|  | | | Healthy | | | | | | | | | | 24.85 (24.45-25.24) | 37.07 (36.67-37.48) | 37.25 (36.88-37.61) | 38.49 (38.10-38.88) | 38.58 (38.17-38.99) | 38.13 (37.38-38.88) | 37.28 (36.52-38.04) | 34.84 (34.06-35.62) | 2.97 (2.84-3.10)^b^ | −1.19 (−1.47 to −0.91)^b^ | −1.22 (−1.31 to −1.13)^b^ |
|  | | | Normal | | | | | | | | | | 27.18 (26.70-27.65) | 41.01 (40.49-41.52) | 44.20 (43.72-44.68) | 45.40 (44.86-45.93) | 45.91 (45.34-46.48) | 45.72 (44.71-46.73) | 44.73 (43.80-45.66) | 43.30 (42.31-44.29) | 4.51 (4.34-4.68)^b^ | −0.88 (−1.26 to −0.51)^b^ | −0.70 (−0.85 to −0.55)^b^ |
|  | | | Unhealthy | | | | | | | | | | 30.26 (29.35-31.16) | 42.70 (41.77-43.63) | 46.19 (45.28-47.10) | 49.76 (48.77-50.74) | 51.55 (50.55-52.56) | 51.69 (50.04-53.33) | 50.63 (49.11-52.16) | 48.45 (46.91-49.98) | 5.23 (4.93-5.53)^b^ | −1.09 (−1.70 to −0.48)^b^ | −1.73 (−1.84 to −1.63)^b^ |
| **Smoking status** | | | | | | | | | | | | | | | | | | | | | | | |
|  | | | Nonsmoker | | | | | | | | | | 25.64 (25.32-25.97) | 38.50 (38.16-38.84) | 38.81 (38.50-39.12) | 39.01 (38.67-39.34) | 38.69 (38.37-39.02) | 39.28 (38.73-39.83) | 38.74 (38.17-39.32) | 36.99 (36.41-37.57) | 2.65 (2.54-2.76)^b^ | −0.56 (−0.77 to −0.34)^b^ | −3.21 (−3.32 to −3.10)^b^ |
|  | | | Smoker | | | | | | | | | | 25.63 (25.12-26.14) | 35.69 (35.15-36.23) | 36.02 (35.48-36.55) | 37.09 (36.48-37.70) | 38.89 (38.19-39.59) | 39.67 (38.18-41.16) | 38.68 (37.16-40.20) | 38.05 (36.52-39.59) | 3.11 (2.92-3.30)^b^ | −0.29 (−0.81 to 0.23) | −3.11 (−3.66 to −2.56)^b^ |
| **Alcohol consumption** | | | | | | | | | | | | | | | | | | | | | | | |
|  | | | 0 days./month | | | | | | | | | | 25.81 (25.50-26.12) | 38.11 (37.78-38.44) | 38.28 (37.97-38.59) | 38.39 (38.06-38.71) | 38.16 (37.84-38.49) | 38.97 (38.42-39.52) | 38.48 (37.91-39.05) | 36.83 (36.25-37.40) | 2.47 (2.37-2.58)^b^ | −0.44 (−0.65 to −0.23)^b^ | −1.43 (−1.74 to −1.12)^b^ |
|  | | | 1-5 days./month | | | | | | | | | | 25.35 (24.77-25.94) | 37.09 (36.45-37.73) | 38.19 (37.54-38.84) | 40.54 (39.80-41.29) | 42.15 (41.40-42.90) | 43.01 (41.45-44.58) | 41.19 (39.58-42.80) | 39.57 (38.01-41.13) | 4.07 (3.85-4.28)^b^ | −0.89 (−1.43 to −0.34)^b^ | −1.22 (−1.31 to −1.13)^b^ |
|  | | | 6-30 days./month | | | | | | | | | | 24.77 (23.90-25.63) | 34.68 (33.67-35.69) | 35.40 (34.28-36.51) | 38.90 (37.54-40.26) | 39.95 (38.55-41.34) | 39.64 (36.62-42.66) | 39.89 (36.96-42.82) | 35.79 (32.93-38.66) | 3.82 (3.46-4.17)^b^ | −1.17 (−2.17 to −0.18)^b^ | −1.44 (−1.60 to −1.28)^b^ |
|  | | | Economic status of households^d^ | | | | | | | | | | | | | | | | | | | | |
|  | | | High | | | | | | | | | | 23.39 (22.47-24.30) | 35.23 (34.27-36.20) | 33.89 (33.03-34.76) | 33.58 (32.82-34.34) | 34.20 (33.48-34.93) | 35.48 (34.19-36.78) | 35.29 (33.90-36.68) | 32.48 (30.99-33.97) | 1.98 (1.72-2.25)^b^ | −0.54 (−1.07 to −0.01)^b^ | −1.92 (−2.06 to −1.78)^b^ |
|  | | | Middle-high | | | | | | | | | | 24.08 (23.62-24.53) | 36.30 (35.76-36.83) | 36.36 (35.90-36.82) | 36.46 (35.98-36.95) | 36.62 (36.14-37.10) | 37.55 (36.67-38.43) | 37.27 (36.38-38.15) | 34.76 (33.89-35.63) | 2.78 (2.62-2.93)^b^ | −0.58 (−0.90 to −0.26)^b^ | −2.92 (−3.20 to −2.65)^b^ |
|  | | | Middle | | | | | | | | | | 25.32 (24.93-25.71) | 36.79 (36.39-37.19) | 37.24 (36.86-37.62) | 38.43 (38.04-38.83) | 38.73 (38.33-39.13) | 39.29 (38.59-39.99) | 38.82 (38.10-39.54) | 38.09 (37.35-38.83) | 2.86 (2.73-2.99)^b^ | −0.23 (−0.50 to 0.04) | −1.26 (−1.51 to −1.02)^b^ |
|  | | | Middle-low | | | | | | | | | | 29.47 (28.79-30.16) | 41.49 (40.85-42.12) | 43.10 (42.46-43.74) | 45.06 (44.32-45.79) | 46.55 (45.72-47.39) | 46.11 (44.67-47.55) | 45.41 (43.82-46.99) | 44.36 (42.76-45.96) | 3.87 (3.63-4.11)^b^ | −0.72 (−1.28 to −0.15)^b^ | −1.29 (−1.43 to −1.15)^b^ |
|  | | | Low | | | | | | | | | | 32.28 (30.94-33.63) | 42.15 (41.04-43.26) | 44.57 (43.43-45.70) | 47.65 (46.29-49.02) | 49.58 (47.88-51.28) | 50.58 (47.45-53.71) | 47.51 (44.30-50.72) | 46.23 (42.83-49.63) | 4.12 (3.66-4.59)^b^ | −1.26 (−2.45 to −0.07)^b^ | −1.06 (−1.17 to −0.96)^b^ |

^a^All βs and β_diff_s were expressed by multiplying 100.

^b^Significant difference (*P*<.05).

^c^BMI was divided into 4 groups according to the 2017 Korean National Growth Charts: underweight (0th-4th percentile), normal (5th-84th percentile), overweight (85th-94th percentile), and obese (95th-100th percentile).

^d^School performance, stress level, and economic status of households were divided into 5 groups: low (0th-19th percentile), middle-low (20th-39th percentile), middle (40th-59th percentile), middle-high (60th-79th percentile), and high (80th-100th percentile).

**Table S3.** Prevalence ratios of self-perceived overweight in socioeconomic factors for each year group participants: 2005-2007, 2008-2010, 2011-2013, 2014-2016, 2017-2019, 2020, 2021, and 2022.

|  | | 2008-2010  versus  2005-2007  (reference), wPR^a^ (95% CI) | *P* value | 2011-2013  versus  2008-2010  (reference), wPR (95% CI) | *P* value | 2014-2016  versus  2011-2013  (reference), wPR (95% CI) | *P* value | 2017-2019  versus  2014-2016  (reference), wPR (95% CI) | *P* value | 2020 versus 2017-2019  (reference), wPR (95% CI) | *P* value | 2021 versus 2020  (reference), wPR (95% CI) | *P* value | 2022 versus 2021  (reference), wPR (95% CI) | *P* value |
| --- | --- | --- | --- | --- | --- | --- | --- | --- | --- | --- | --- | --- | --- | --- | --- |
| Overall | | 1.19 (1.18-1.21)^b^ | <.001^b^ | 1.01 (0.98-1.03) | .637 | 1.01 (0.97-1.05) | .647 | 1.00 (0.96-1.05) | .975 | 1.01 (0.93-1.09) | .808 | 0.99 (0.88-1.11) | .869 | 0.97 (0.85 -1.12) | .706 |
| **Grade** | | | | | | | | | | | | | | | |
|  | 7th-9th grade (middle school) | 1.20 (1.18-1.23)^b^ | <.001^b^ | 0.99 (0.96-1.02) | .543 | 0.98 (0.94-1.02) | .400 | 1.01 (0.95-1.06) | .833 | 1.04 (0.94-1.15) | .428 | 0.99 (0.85-1.15) | .892 | 0.97 (0.81-1.16) | .731 |
|  | 10th-12th grade (high school) | 1.18 (1.15-1.21)^b^ | <.001^b^ | 1.02 (0.98-1.07) | .312 | 1.03 (0.98-1.09) | .264 | 0.99 (0.93-1.06) | .878 | 0.98 (0.87-1.11) | .788 | 0.99 (0.84-1.17) | .929 | 0.98 (0.80-1.21) | .850 |
| **Sex** | | | | | | | | | | | | | | | |
|  | Male | 1.14 (1.12-1.16)^b^ | <.001^b^ | 0.99 (0.96 -1.01) | .373 | 1.01 (0.98-1.05) | .551 | 1.03 (0.98-1.08) | .270 | 1.05 (0.95-1.16) | .342 | 1.00 (0.86-1.17) | .961 | 0.97 (0.82-1.15) | .744 |
|  | Female | 1.26 (1.23-1.29)^b^ | <.001^b^ | 1.03 (0.99-1.07) | .120 | 1.00 (0.95-1.06) | .864 | 0.97 (0.91-1.04) | .349 | 0.97 (0.86-1.09) | .574 | 0.98 (0.83-1.15) | .773 | 0.98 (0.80-1.19) | .805 |
| **Region of residence** | | | | | | | | | | | | | | | |
|  | Urban | 1.19 (1.17-1.21)^b^ | <.001^b^ | 1.01 (0.98-1.03) | .682 | 1.01 (0.97-1.05) | .746 | 1.00 (0.96-1.05) | .986 | 1.01 (0.93-1.10) | .811 | 0.99 (0.88 -1.11) | .845 | 0.97 (0.84-1.12) | .712 |
|  | Rural | 1.21 (1.16-1.25) | <.001^b^ | 1.02 (0.95-1.09) | .611 | 1.05 (0.93-1.18) | .452 | 1.01 (0.84-1.21) | .939 | 1.00 (0.77-1.32) | .971 | 1.03 (0.72-1.50) | .857 | 0.97 (0.63-1.51) | .908 |
| **BMI group^c^** | | | | | | | | | | | | | | | |
|  | Underweight | 1.01 (1.00-1.01) | .159 | 1.00 (0.99-1.01) | .784 | 1.00 (0.99-1.02) | .646 | 1.00 (0.98-1.02) | .804 | 1.00 (0.96-1.03) | .916 | 1.00 (0.95-1.04) | .830 | 1.00 (0.96-1.05) | .939 |
|  | Normal | 1.22 (1.20-1.24)^b^ | <.001^b^ | 0.99 (0.96-1.02) | .409 | 0.98 (0.94-1.01) | .226 | 0.96 (0.92-1.00)^b^ | .048^b^ | 0.99 (0.92-1.06) | .789 | 0.98 (0.89 -1.08) | .660 | 0.99 (0.89-1.11) | .929 |
|  | Overweight | 2.53 (1.97-3.25)^b^ | <.001^b^ | 0.84 (0.55-1.29) | .426 | 0.75 (0.45-1.22) | .244 | 0.70 (0.42-1.15) | .157 | 0.77 (0.38-1.60) | .489 | 0.98 (0.38 -2.58) | .974 | 1.17 (0.32-4.32) | .815 |
|  | Obese | 2.22 (1.09-4.54)^b^ | .029^b^ | 0.72 (0.30-1.71) | .458 | 1.00 (0.38-2.65) | .999 | 0.72 (0.26-2.06) | .545 | 0.86 (0.22-3.42) | .834 | 1.05 (0.16 -6.98) | .959 | 0.85 (0.09 -8.11) | .889 |
| **School performance^d^** | | | | | | | | | | | | | | | |
|  | High | 1.18 (1.14-1.22)^b^ | <.001^b^ | 0.98 (0.93-1.04) | .591 | 0.98 (0.92-1.06) | .683 | 1.00 (0.92 -1.10) | .946 | 1.02 (0.86 -1.20) | .823 | 0.98 (0.78-1.25) | .895 | 0.97 (0.73-1.29) | .853 |
|  | Middle-high | 1.19 (1.17-1.22)^b^ | <.001^b^ | 1.00 (0.96-1.04) | .915 | 1.01 (0.95-1.06) | .823 | 0.99 (0.92-1.06) | .788 | 0.99 (0.87-1.14) | .930 | 1.00 (0.82-1.22) | .991 | 0.98 (0.78-1.22) | .830 |
|  | Middle | 1.18 (1.15-1.20)^b^ | <.001^b^ | 1.01 (0.97-1.05) | .725 | 1.01 (0.95-1.06) | .843 | 1.01 (0.93-1.08) | .888 | 1.02 (0.90 -1.16) | .785 | 0.98 (0.82-1.18) | .865 | 0.97 (0.79-1.19) | .772 |
|  | Middle-low | 1.21 (1.17-1.24)^b^ | <.001^b^ | 1.01 (0.96-1.06) | .723 | 1.03 (0.96-1.10) | .372 | 1.01 (0.93-1.10) | .826 | 1.01 (0.87-1.18) | .880 | 0.99 (0.79-1.26) | .961 | 0.98 (0.75-1.30) | .906 |
|  | Low | 1.16 (1.11-1.21)^b^ | <.001^b^ | 1.03 (0.96-1.10) | .406 | 1.05 (0.95-1.15) | .356 | 1.02 (0.90-1.17) | .740 | 0.99 (0.76 -1.29) | .935 | 1.01 (0.70-1.45) | .976 | 0.98 (0.64-1.50) | .919 |
| **Stress level^d^** | | | | | | | | | | | | | | | |
|  | High | 1.23 (1.18-1.29)^b^ | <.001^b^ | 1.02 (0.95-1.09) | .638 | 1.02 (0.92 -1.14) | .648 | 1.00 (0.87-1.15) | .977 | 1.01 (0.76-1.34) | .932 | 0.96 (0.65-1.42) | .843 | 0.97 (0.65-1.44) | .863 |
|  | Middle-high | 1.21 (1.19 -1.24)^b^ | <.001^b^ | 1.02 (0.98-1.07) | .370 | 1.03 (0.97-1.10) | .311 | 0.99 (0.92-1.07) | .769 | 1.01 (0.87-1.18) | .872 | 0.97 (0.79-1.20) | .805 | 0.96 (0.76-1.23) | .770 |
|  | Middle | 1.19 (1.17-1.22)^b^ | <.001^b^ | 1.01 (0.98 -1.04) | .524 | 1.01 (0.97-1.05) | .696 | 1.00 (0.95-1.06) | .977 | 1.02 (0.91-1.14) | .724 | 0.98 (0.84-1.15) | .811 | 0.98 (0.82-1.18) | .831 |
|  | Middle-low | 1.15 (1.12 -1.19)^b^ | <.001^b^ | 0.99 (0.95-1.04) | .693 | 1.02 (0.95-1.08) | .608 | 1.00 (0.92-1.08) | .992 | 1.03 (0.89-1.19) | .730 | 1.00 (0.80-1.25) | .967 | 0.96 (0.73-1.26) | .748 |
|  | Low | 1.15 (1.07-1.23)^b^ | <.001^b^ | 0.96 (0.86-1.07) | .453 | 1.03 (0.91-1.16) | .694 | 1.00 (0.85-1.18) | .979 | 1.01 (0.76-1.35) | .948 | 1.02 (0.65-1.61) | .931 | 0.97 (0.56-1.67) | .900 |
| **Subjective health status** | | | | | | | | | | | | | | | |
|  | Very healthy | 1.19 (1.1-1.22)^b^ | <.001^b^ | 1.00 (0.97-1.03) | .861 | 1.02 (0.98-1.07) | .383 | 1.00 (0.94-1.06) | .960 | 0.99 (0.89-1.11) | .899 | 0.99 (0.84-1.15) | .862 | 0.96 (0.80-1.16) | .683 |
|  | Healthy | 1.23 (1.20-1.27)^b^ | <.001^b^ | 1.06 (1.01-1.11)^b^ | .015^b^ | 1.02 (0.96-1.09) | .496 | 1.01 (0.92-1.10) | .835 | 1.00 (0.84 -1.18) | .969 | 0.98 (0.78-1.23) | .875 | 0.97 (0.76-1.25) | .839 |
|  | Normal | 1.22 (1.16-1.28)^b^ | <.001^b^ | 1.06 (0.98-1.16) | .155 | 1.07 (0.94-1.21) | .284 | 1.04 (0.87-1.24) | .689 | 1.00 (0.73-1.38) | .987 | 0.98 (0.65-1.48) | .919 | 0.96 (0.60-1.53) | .856 |
|  | Unhealthy | 1.21 (1.19-1.23)^b^ | <.001^b^ | 1.01 (0.98-1.03) | .728 | 1.00 (0.96-1.04) | .872 | 0.99 (0.95-1.04) | .830 | 1.01 (0.93-1.10) | .819 | 0.99 (0.88-1.11) | .882 | 0.97 (0.84-1.12) | .703 |
| **Smoking status** | | | | | | | | | | | | | | | |
|  | Nonsmoker | 1.16 (1.13-1.18)^b^ | <.001^b^ | 1.01 (0.96-1.05) | .821 | 1.02 (0.95-1.08) | .604 | 1.03 (0.93-1.14) | .563 | 1.01 (0.82-1.26) | .907 | 0.98 (0.71-1.36) | .922 | 0.99 (0.69-1.43) | .957 |
|  | Smoker | 1.20 (1.18-1.22)^b^ | <.001^b^ | 1.00 (0.98-1.03) | .844 | 1.00 (0.96-1.04) | .927 | 1.00 (0.95-1.04) | .876 | 1.01 (0.93-1.10) | .756 | 0.99 (0.88-1.11) | .892 | 0.97 (0.85-1.12) | .712 |
| **Alcohol consumption** | | | | | | | | | | | | | | | |
|  | 0 days/month | 1.19 (1.15-1.22)^b^ | <.001^b^ | 1.02 (0.96-1.07) | .523 | 1.04 (0.96-1.12) | .331 | 1.03 (0.92-1.15) | .643 | 1.02 (0.80-1.29) | .901 | 0.97 (0.68-1.39) | .864 | 0.97 (0.66-1.44) | .892 |
|  | 1-5 days/month | 1.15 (1.10-1.20)^b^ | <.001^b^ | 1.01 (0.93-1.10) | .804 | 1.06 (0.92-1.22) | .436 | 1.02 (0.82-1.26) | .872 | 0.99 (0.65-1.53) | .982 | 1.00 (0.53-1.91) | .990 | 0.94 (0.47-1.88) | .853 |
|  | 6-30 days/month | 1.18 (1.14-1.23)^b^ | <.001^b^ | 0.98 (0.92-1.05) | .553 | 1.00 (0.91-1.08) | .912 | 1.01 (0.91-1.12) | .863 | 1.02 (0.85-1.23) | .837 | 1.00 (0.77-1.29) | .982 | 0.96 (0.69-1.33) | .800 |
| **Economic status of households^b^** | | | | | | | | | | | | | | | |
|  | High | 1.19 (1.16-1.22)^b^ | <.001^b^ | 1.00 (0.96-1.04) | .962 | 1.00 (0.95-1.06) | .954 | 1.00 (0.94-1.07) | .946 | 1.01 (0.89-1.15) | .819 | 1.00 (0.83-1.20) | .962 | 0.96 (0.78-1.19) | .714 |
|  | Middle-high | 1.18 (1.16-1.20)^b^ | <.001^b^ | 1.01 (0.98-1.04) | .664 | 1.02 (0.97-1.07) | .410 | 1.00 (0.95-1.06) | .867 | 1.01 (0.91-1.12) | .863 | 0.99 (0.85-1.15) | .920 | 0.99 (0.82-1.19) | .899 |
|  | Middle | 1.21 (1.17-1.25)^b^ | <.001^b^ | 1.03 (0.97-1.09) | .343 | 1.04 (0.95-1.13) | .415 | 1.03 (0.91-1.17) | .671 | 0.99 (0.77-1.27) | .948 | 0.99 (0.69-1.42) | .944 | 0.98 (0.63-1.52) | .933 |
|  | Middle-low | 1.17 (1.10-1.25)^b^ | <.001^b^ | 1.04 (0.94-1.16) | .437 | 1.06 (0.90-1.24) | .487 | 1.04 (0.80-1.35) | .781 | 1.02 (0.59-1.78) | .944 | 0.94 (0.43-2.08) | .882 | 0.98 (0.38 -2.50) | .960 |
|  | Low | 1.19 (1.16-1.22)^b^ | <.001^b^ | 1.00 (0.96-1.04) | .962 | 1.00 (0.95-1.06) | .954 | 1.00 (0.94-1.07) | .946 | 1.01 (0.89-1.15) | .819 | 1.00 (0.83-1.20) | .962 | 0.96 (0.78-1.19) | .714 |

^a^wPR: weighted prevalence ratio.

^b^Significant difference (*P*<.05).

^c^BMI was divided into 4 groups according to the 2017 Korean National Growth Charts: underweight (0th-4th percentile), normal (5th-84th percentile), overweight (85th-94th percentile), and obese (95th-100th percentile).

^d^School performance, stress level, and economic status of households were divided into 5 groups: low (0th-19th percentile), middle-low (20th-39th percentile), middle (40th-59th percentile), middle-high (60th-79th percentile), and high (80th-100th percentile).
