# Supplementary figures and images for: National Trends in the Prevalence of Self-Perceived Overweight Among Adolescents Between 2005 and 2022: Nationwide Representative Study
Source: JMIR Public Health Surveill. 2024 Oct 9;10:e57803. doi: 10.2196/57803 (PMC11499719; doi:10.2196/57803)

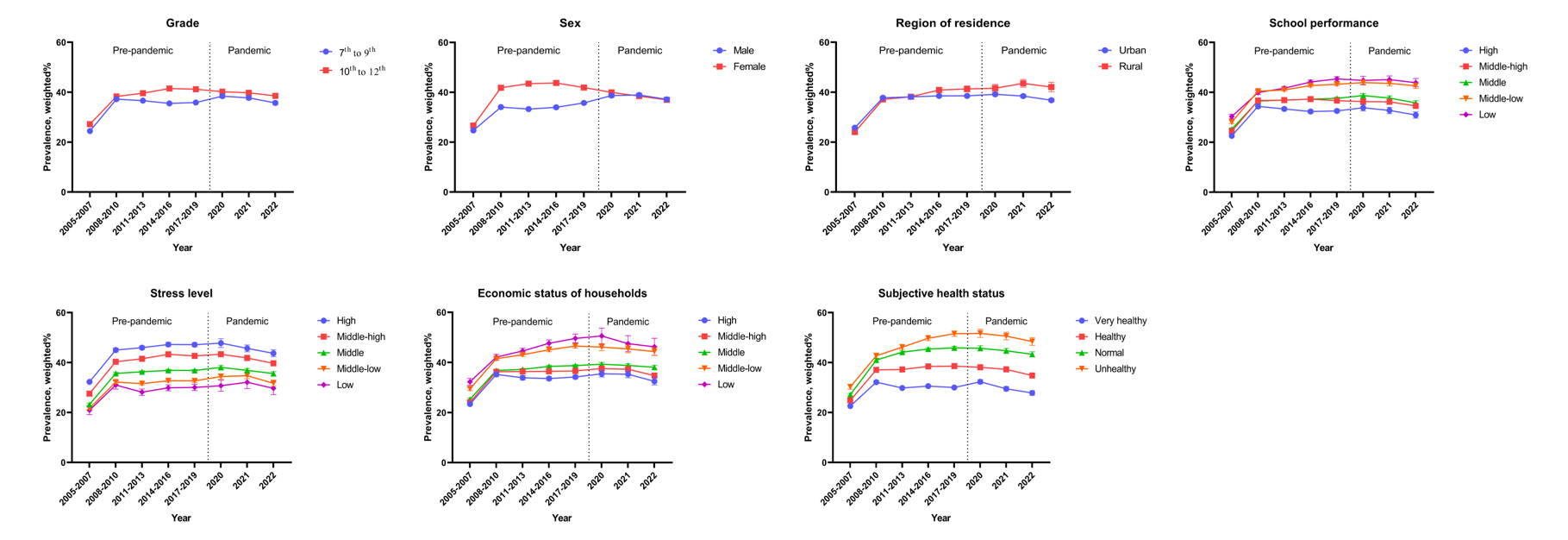

Supplement: Multimedia Appendix 2 [file publichealth_v10i1e57803_app2.png]
